# CCS2, an Octatricopeptide-Repeat Protein, Is Required for Plastid Cytochrome *c* Assembly in the Green Alga *Chlamydomonas reinhardtii*

**DOI:** 10.3389/fpls.2017.01306

**Published:** 2017-08-03

**Authors:** Sara G. Cline, Isaac A. Laughbaum, Patrice P. Hamel

**Affiliations:** ^1^Department of Molecular Genetics and Department of Biological Chemistry and Pharmacology, The Ohio State University, Columbus OH, United States; ^2^Plant Cellular and Molecular Biology Graduate Program, The Ohio State University, Columbus OH, United States

**Keywords:** plastid, photosynthesis, cytochrome *c*, heme, assembly factor, OPR

## Abstract

In bacteria and energy generating organelles, *c*-type cytochromes are a class of universal electron carriers with a heme cofactor covalently linked via one or two thioether bonds to a heme binding site. The covalent attachment of heme to apocytochromes is a catalyzed process, taking place via three evolutionarily distinct assembly pathways (Systems I, II, III). System II was discovered in the green alga *Chlamydomonas reinhardtii* through the genetic analysis of the *ccs* mutants (**c**ytochrome ***c*s**ynthesis), which display a block in the apo- to holo- form conversion of cytochrome *f* and *c*_6_, the thylakoid lumen resident *c*-type cytochromes functioning in photosynthesis. Here we show that the gene corresponding to the *CCS2* locus encodes a 1,719 amino acid polypeptide and identify the molecular lesions in the *ccs2-1* to *ccs2-5* alleles. The CCS2 protein displays seven degenerate amino acid repeats, which are variations of the **o**ctatrico**p**eptide-**r**epeat motif (OPR) recently recognized in several nuclear-encoded proteins controlling the maturation, stability, or translation of chloroplast transcripts. A plastid site of action for CCS2 is inferred from the finding that GFP fused to the first 100 amino acids of the algal protein localizes to chloroplasts in *Nicotiana benthamiana*. We discuss the possible functions of CCS2 in the heme attachment reaction.

## Introduction

Energy-transducing membranes are specialized membranes in archaea, bacteria, mitochondria, and chloroplasts, which rely on electron carriers to generate the proton gradient necessary for ATP synthesis. In energy-transducing membranes, the *c*-type cytochromes, also generically referred to as cytochromes *c*, are a class of structurally diverse metalloproteins with one or more covalently linked heme(s) (ferro-protoporphyrin IX) that participate in electron transfer reactions (Thony-Meyer, 1997; Bonnard et al., 2010; Verissimo and Daldal, 2014). On the positive side (*p*-side) of the energy-transducing membrane (i.e., bacterial or archaeal periplasm, mitochondrial intermembrane space, thylakoid lumen), a group of cytochromes *c* occur as both soluble and membrane proteins with the heme moiety typically attached *via* a thioether linkage to two cysteine sulfhydryl(s) in a characteristic motif in the apocytochrome *c*. The consensus motif, also referred to as the heme-binding site, is CX_n_CH where n is usually equal to two and X can be any residue except cysteine in naturally occurring cytochromes *c*. The histidine in this motif acts as a ligand of ferroheme (Bowman and Bren, 2008; Allen et al., 2009). Heme attachment to the heme-binding site is a catalyzed reaction *in vivo* and requires heme transport across at least one biological membrane and covalent linkage of ferroheme to free sulfhydryls of the heme binding cysteines in the CX_n_CH motif (Bonnard et al., 2010; Mavridou et al., 2013).

To date, three Systems (I, II, and III) have been identified as being necessary for the post-translational assembly of cytochromes *c* located on the *p*-side of the membrane, where these molecules function. These systems have been identified through extensive investigation of both bacterial and eukaryotic models (Kranz et al., 2009; Allen, 2011; de Vitry, 2011; Mavridou et al., 2013; Verissimo and Daldal, 2014). In plastids, the heme attachment reaction occurs on the luminal side of the thylakoid membrane and is under the control of System II. This system is composed of multiple pathways and occurs in plastids of all photosynthetic eukaryotes, cyanobacteria, and most proteobacteria of the β-, δ-, and 𝜀-group (Hamel et al., 2009; Bonnard et al., 2010; Simon and Hederstedt, 2011).

System II first emerged through genetic screens for photosynthesis-impaired *ccs* mutants (*ccs* for **c**ytochrome ***c*** **s**ynthesis) in the green alga *Chlamydomonas reinhardtii* (Hamel et al., 2009; Simon and Hederstedt, 2011; Gabilly and Hamel, 2017). The *ccs* mutants were isolated on the basis of photosynthetic deficiency due to loss of plastid *c*-type cytochromes, namely membrane-bound cytochrome *f* and soluble cytochrome *c*_6_ (Howe and Merchant, 1992). These two plastid cytochromes *c* reside in the thylakoid lumen and function in photosynthesis. The defect is specific to plastid cytochromes *c*, as abundance of mitochondrial cytochromes *c* was unaffected in the *ccs* mutants (Howe and Merchant, 1992). Pulse-chase analyses revealed that precursor forms of apocytochrome *f* and *c*_6_ are synthesized, translocated to the lumen, and therein cleaved by the thylakoid peptidase. However, they are not converted to their respective holoforms. This indicates that the *CCS* loci control the heme attachment reaction in the lumen (Howe and Merchant, 1992, 1993, 1994; Xie et al., 1998; Gabilly et al., 2010). At least seven loci, plastid *ccsA* and nuclear *CCS1* to *CCS6*, were uncovered through genetic analysis of the *ccs* mutants isolated via several UV and insertional mutageneses (Xie et al., 1998; Page et al., 2004; Gabilly et al., 2010).

Functional analysis of plastid CcsA and Ccs1 led to the proposal that these two proteins act together to relay heme from its site of synthesis, the stroma, to its site of function, the lumen, possibly in an assembly complex alongside other CCS factors (Xie and Merchant, 1996; Inoue et al., 1997; Dreyfuss et al., 2003; Hamel et al., 2003). Biochemical studies supported this model with evidence that bacterial CcsA and Ccs1 operate as a functional unit with a dual activity in heme transport across the membrane and heme ligation to apocytochromes *c* in the periplasmic space (Frawley and Kranz, 2009).

In addition to heme delivery and attachment, the maintenance of the cysteine sulfhydryls (reduced vs. oxidized) within the heme binding site is a prerequisite for covalent linkage of heme (Bonnard et al., 2010; Sanders et al., 2010; Verissimo and Daldal, 2014). The proposed model is that the cysteines are first oxidized by disulfide bond forming enzymes on the *p*-side of the membrane and must subsequently be reduced to provide free sulfhydryls for the heme ligation reaction. In plastids and bacteria, this requirement is fulfilled by the operation of a disulfide reducing pathway, which conveys electrons across the energy-transducing membrane via thiol-disulfide exchanges in order to reduce the disulfide bond formed between the heme-linking cysteines in the heme binding motif of apocytochromes *c* (Karamoko et al., 2013; Verissimo and Daldal, 2014; Gabilly and Hamel, 2017).

In bacteria, the CcsA/Ccs1-dependent heme delivery/attachment pathway and the disulfide-reducing pathway are all that is required for cytochrome *c* assembly (Beckett et al., 2000; Le Brun et al., 2000; Feissner et al., 2005). However, screening of *ccs* mutants in *Chlamydomonas reinhardtii* has revealed four additional *CCS* loci (*CCS2*, *CCS3*, *CCS4*, and *CCS6*), an indication that the assembly of cytochromes *c* in the plastid is a more complicated process (Xie et al., 1998; Page et al., 2004). While we have proposed that CCS4 regulates the disulfide reducing pathway in the plastid (Gabilly et al., 2011), the gene products for *CCS2*, *CCS3*, and *CCS6* remain unknown.

In this article, we report the molecular identification of the *CCS2* locus by functional complementation of the *ccs2* mutant. The *CCS2* gene encodes a plastid-localized, 170 kDa protein that was previously identified by bioinformatics to be a member of the **o**ctatrico**p**eptide **r**epeat (OPR) family (Eberhard et al., 2011).

## Materials and Methods

### Strains and Culture Condition

The *ccs2-1* through *ccs2-5* strains were used in complementation experiments (Xie et al., 1998). Mutants *ccs2-1* and *ccs2-2* were crossed to wild-type strain 3A^+^ (*mt^+^ arg7-8*) and 4C^-^ (*mt*^-^ *arg7-8*), respectively, to generate the *ccs2-1 arg7-8* and *ccs2-2 arg7-8* recipient strains. The *ccs2-1 arg7-8* mutant was then crossed to wild-type strain CC425 (*arg7-8 cw15*) to generate the *ccs2-1 arg7-8 cw15* mutant used for detection of the HA-tagged CCS2 protein. The MCA1-HA expressing strain is described in Raynaud et al. (2007). Strains were maintained at 25°C on Tris-acetate phosphate (TAP) liquid or solid medium supplemented with 400 mg/mL arginine (Harris, 1989) at 0.6 μE/m^2^/s. Complemented *ccs2* strains were assessed for restoration of photoautotrophic growth on minimal medium (Min) (Harris, 1989) or acetate containing (1.7 mM) minimal medium. For protein extraction, wild-type and complemented strains were grown in liquid TAP supplemented with arginine under 50 μE/m^2^/s illumination while *ccs* mutants were grown under 0.6 μE/m^2^/s illumination. Cell wall-less mutants (*cw15*) were cultured in liquid and on solid media supplemented with 50 mM sorbitol. Copper-free media for induction of cytochrome *c*_6_ was prepared as described previously (Howe and Merchant, 1992; Quinn and Merchant, 1998).

### Molecular Cloning of the *CCS2* Gene

An indexed cosmid library of *Chlamydomonas* genomic DNA was used for transformation by electroporation as described by Shimogawara et al. (1998) with the following exceptions: the vector backbone was the cosmid pCB412 containing the *ARG7* marker and the transformation required a 30 min incubation in autolysin (to digest the cell wall) followed by electroporation of 5 μg of DNA per cosmid pool on a Biorad Micropulser at 1300 V. Transformants were selected under 50 μE/m^2^/s light on minimal media supplemented with 1.7 mM acetate. Plasmid pMOL+8.2 kb was generated by cloning the 8.2 kb *Bam*HI/*Hind*III *CCS2* genomic fragment isolated from complementing cosmid (c8G6), into *Bam*HI/*Hind*III digested pMOLUC (Cha et al., 2002).

### Assembly of the *CCS2* cDNA

*Chlamydomonas* RNAs were extracted and retro-transcribed using a bacterial reverse transcriptase from the Roche Transcriptor High Fidelity cDNA Synthesis Kit and the *CCS2* specific primers CCS2.30 and CCS2.66STP. Overlapping fragments were amplified with the following primer pairs: CCS2.69 and CCS2.66STP, CCS2.19 and CCS2.54, CCS2.21 and CCS2.18, CCS2.79 and CCS2.02, CCS2.27 and CCS2.28, CCS2.51 and CCS2.26, and, finally, CCS2.81ATG and CCS2.70 using DV Ready Mix (Sigma) and the total retro-transcribed RNAs as a template. Amplified fragments were isolated after electrophoresis in agarose gel, purified, re-amplified and AT-cloned into pGEM-T Easy for sequencing. All primer sequences are listed in Supplementary Table S1.

### Construction of HA-Tagged CCS2 and CCS2-GFP Expressing Constructs

Versions of the *CCS2* gene carrying an internal HA tag were created by cutting pMOL+8.2 kb by *Bsi*WI or *Bsp*EI and inserting the HA-tag sequence via In-Fusion^®^ (CloneTech). The HA-tag sequence was generated using PCR based fill-in of primers CCS2-HA_BsiW1-F and CCS2-HA_BsiW1-R or CCS2-HA_BspEI-F and CCS2-HA_BspEI-R. Introduction of an internal HA tag sequence at the *Bsp*EI site (residue 298) abolished CCS2 function while the tag at *Bsi*WI (residue 1672) had no impact (not shown).

To generate the series of “p8” plasmids used in **Figure 5**, a 606 bp sequence was synthesized (Genscript) and cloned into pUC57 (pUC57+ccs2bit). This sequence contained two distinct fragments that were modified from the *Chlamydomonas* genomic DNA. The first was a 207 bp fragment corresponding to the 3′ end of *CCS2* genomic DNA that both changed the stop codon in the *CCS2* ORF into an alanine and added the restriction sites *Xba*I, *Swa*I, and *Spe*I downstream of the stop codon. The second was a 394 bp fragment encompassing the 5′ end of *CCS2* genomic DNA designed to remove the native *CCS2* promoter and introduce restriction sites *Bgl*II and *Xho*I upstream of the ATG.

Carboxy-terminus 3xHA tags were generated by cutting pMOL+8.2 kb by *Bsi*WI (146 bp upstream the stop codon) and *PshA*1 (570 bp downstream of the stop codon in the 3′ UTR) and cloning the 207 bp fragment, which was amplified by primers CCS2-BsiWI-F and CCS2-PshAI-R using pUC57+ccs2bits as a template. The 207 bp fragment was cloned using In-Fusion^®^ and the resulting plasmid is p8-noS. The p8-noS plasmid was then digested by *Xba*I and *Spe*I and a sequence corresponding to a 3xHA tag (and including a stop), created through PCR fill-in of primers 8.3xHA-f and 8.3xHA-r, was inserted via In-Fusion^®^. This created vector p8-3xHA expressing CCS2-HA from its native promotor. The plasmids expressing CCS2 or CCS2-HA under the *PSAD* promoter were generated in a similar manner. The 394 bp fragment was amplified from pUC57+ccs2bits using primers CCS2-XcmI.R and CCS2-XcmI.F and inserted via In-Fusion^®^ after digestion of pMOL+8.2 kb and p8-3xHA by *Xcm*I (322 bp from the initiation codon). This created plasmids p8-noP and p8-3xHAnoP. These constructs were then used to generate plasmids p8-PROM and p8-3xHA+P. To create these vectors, the *PSAD* promoter and 5′ UTR were amplified from plasmid pSL18 (Pollock et al., 2004), using primers 8.PROM-f and 8.PROM-r, and inserted via In-Fusion^®^ at the introduced restriction sites *Bgl*II and *Xho*I.

The plasmid pGWB5/ccs2target, expressing the CCS2-GFP fusion protein, was constructed from pUC57+ccs2targeting, which contains the first 300 bp from *CCS2* ORF, corresponding to the first 100 amino acids of CCS2, cloned into pUC57. This 300 bp sequence was codon optimized for expression in tobacco and synthesized by GenScript. Using primers CCS2.t1 and CCS2.t2, the sequence was amplified from the pUC57+ccs2targeting template and then inserted in frame with the GFP reporter in the expression vector pGWB5 (Nakagawa et al., 2007) using entry vector pENTR/SD/TOPO (Invitrogen) via TOPO cloning. The GFP-NLS/GFP-NES expressing construct in pK7WGF2 (Karimi et al., 2002) is a gift from Dr. I. Meier (Ohio State University). The NLS (nuclear localization sequence) is from the SV40 large T-antigen. The NES (nuclear export signal) is from the HIV-1 Rev response element. Plasmids were transferred to *Agrobacterium tumefaciens* GV3101 by electroporation.

### Protein Preparation and Analysis

Cytochrome *f* and *c*_6_ detection was performed as in Howe and Merchant (1992). Soluble fractions for cytochrome *c*_6_ detection were obtained by freeze-thaw fractionation of cells grown in copper deficient conditions. Fractions were electrophoretically separated and cytochromes *c* revealed by immunodetection or a heme-staining procedure (Howe and Merchant, 1992). Anti-HA immunoblotting analysis was performed on whole cells extracts, prepared as follows: cells were grown to early logarithmic phase under 0.6 μE/m^2^/s illumination and moved into 30 μE/m^2^/s light for 5 h. Cells were then pelleted and re-suspended in 10 mM NaPO_4_ buffer with protease inhibitors (4 mM benzamine, 0.4 mM 6-amino-*n*-hexanoic acid, 2 mM PMSF, 10 μM leupeptin, 1 μM pepstatin, 1 mM ortho-phenanthroline, 40 μg/mL chymostatin, 10 μM E-64) (Kurvari et al., 1998) to a final concentration of 3 × 10^8^ cells. Laemmli Buffer was then added to a final concentration of 1.2 × 10^8^ cells and 0.1 M DTT. Cells were placed in a sonication bath for 120 s and the solubilized protein sample was denatured for 20 min at 70°C before electrophoresis. Polyclonal antisera raised against *Chlamydomonas* cytochrome *c*_6_, cytochrome *f* GST-fusion protein, and CF_1_ were used for immunodetection by alkaline phosphatase-conjugated secondary antibodies. The rat monoclonal anti-HA antibody (clone 3F10) (Roche) was used for immunodetection by peroxidase-conjugated secondary antibody.

### Fluorescence Rise and Decay Kinetics

Fluorescence rise and decay kinetics were taken as described in Gabilly et al. (2010) except that strains were grown in liquid medium overnight and measurements were recorded on 20 μL culture aliquots against a white background. Fluorescence transients were measured using Handy Fluorcam from Photon System Instruments. The fluorescence is in arbitrary units (A.U.) and recorded over a 3-s illumination period.

### GFP Fluorescence and Imaging

*Nicotiana benthamiana* was transformed with *Agrobacterium* carrying the GFP-CCS2 or NLS-GFP/NES-GFP expressing construct *via* infiltration after a 1 h incubation in Induction Medium (10 mM MgCl_2_, 10 mM 2-[*N*-Morpholino]ethanesulfonic acid, 100 μM Acetosyringone). After 3 days, protoplasts were extracted from infiltrated leaves by a 30-min incubation in a Digestion Buffer (1.5% cellulose, 0.4% macerozyme, 0.4 M mannitol, 20 mM KCl, 20 mM 2-[*N*-Morpholino]ethanesulfonic acid -KOH pH 5.5, 10 mM CaCl_2_, 0.1% Bovine serum albumin) and concentrated with a 1 s spin at 100 rpm. Supernatant was removed and protoplasts were re-suspended in 100 μL Digestion Buffer before imaging. GFP-dependent fluorescence was taken at 515 nm and chlorophyll auto-fluorescence at 650 nm on a Nikon Cl confocal microscope (Eclipse C90i) using a medium aperture. Images were processed using the NIS-Elements software.

### Analysis of OPR Motifs in Proteins

Information presented in **Figure 4** was generated by the MEME algorithm (Bailey and Elkan, 1994). Individual protein sequences (Supplementary Figure S1) were entered into the program and base settings were altered to search for any number of repetitions, one motif, between 70 and 600 sites, and between 28 and 50 amino acids wide. The OPR motifs identified by the MEME program (Supplementary Figure S2) were fed into Weblogo 3.3 (Crooks et al., 2004).

## Results

### Cloning of the *CCS2* Gene by Functional Complementation of the *ccs2-2* Mutant

**FIGURE 1:**
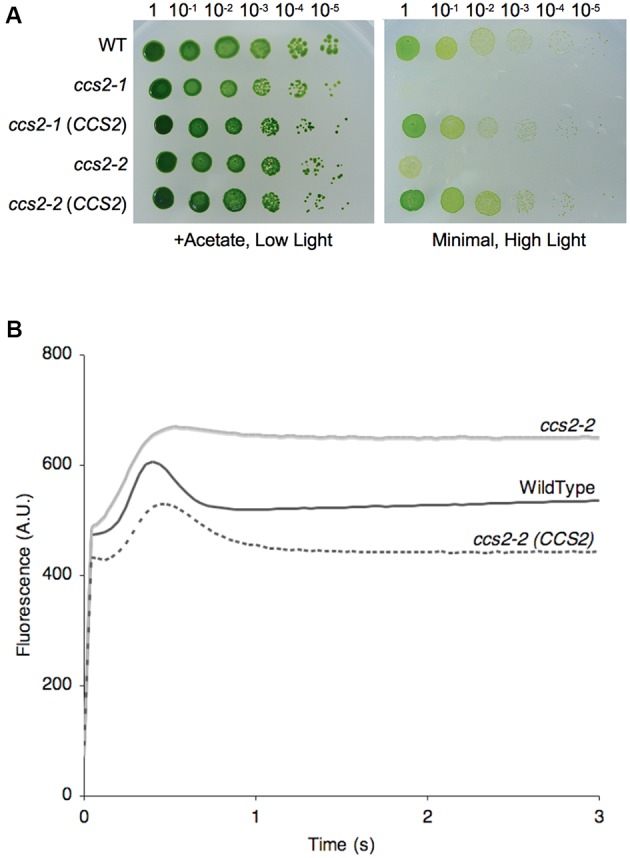
The *CCS2* gene restores photoautotropic growth to the *ccs2* mutants. For **(A,B)**, wild-type CC124 (WT), *ccs2-1* and *ccs2-2* strains, and *CCS2* complemented mutants were used. **(A)** Ten-fold dilution series of algal cultures were plated on solid medium, with or without acetate, and incubated under low light (0.6 μE/m^2^/s) or high light (50 μE/m^2^/s), respectively, for 1 week at 25°C. The *ccs2-3*, *ccs2-4*, and *ccs2-5* mutants are also restored for photoautotrophic growth upon transformation with the *CCS2* gene (not shown). **(B)** Representative fluorescence rise and decay kinetics indicate restoration of the cytochrome *b*_6_*f* complex in the complemented *ccs2* mutant. While all complemented mutants were restored, only the *ccs2-2* strain is shown. The fluorescence is in arbitrary units (A.U.) and recorded over a 3-s illumination period after a dark adaption period.

To gain further insights into plastid cytochrome *c* assembly, we sought to clone the gene corresponding to the *CCS2* locus, defined by the *ccs2-1* to *-5* alleles (Xie et al., 1998). All *ccs* mutants display a *b*_6_*f*-deficient phenotype due to loss of cytochrome *f* assembly and hence are unable to grow photoautotrophically (Howe and Merchant, 1992; Xie et al., 1998). Using the photosynthetic deficient phenotype of the *ccs* mutants, we cloned the *CCS2* gene via transformation of the *ccs2-2 arg7-8* double mutant with an indexed cosmid library (Purton and Rochaix, 1994; Zhang et al., 1994). Transformants were selected for restored growth on acetate-containing minimal medium under standard illumination (50 μE/m^2^/s). Two cosmids (c8G6 and c5D9) were isolated based on their ability to restore photosynthetic growth to the *ccs2-2* mutant. Sequence analysis revealed that c8G6 contains a 30.2 kb region from chromosome 19 while c5D9 appears to have rearranged. The complementing activity in c8G6 could be narrowed down to an 8.2 kb *Bam*HI-*Hind*III fragment. Both c8G6 and the cloned 8.2 kb *Bam*HI-*Hind*III fragment complemented strains *ccs2-1* through *ccs2-5*, restoring photosynthetic growth (**Figure 1A**).

**FIGURE 2:**
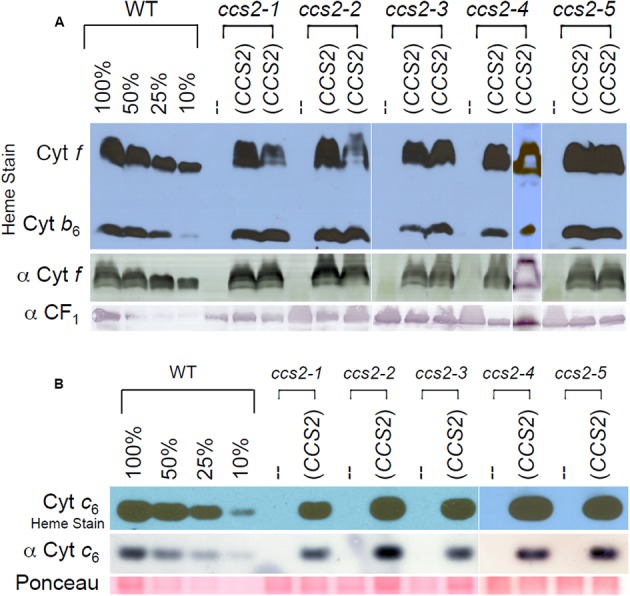
*Restoration of plastid cytochrome c* assembly in the *CCS2* complemented strains. For **(A,B)**, the *ccs2-1* to *ccs2-5* mutants were complemented with the 8.2 kb genomic fragment containing the *CCS2* gene. Dilutions of the wild-type sample serve to estimate the cytochrome *f* **(A)** and cytochrome *c*_6_ **(B)** abundance. **(A)** The wild-type CC124 strain (WT), *ccs2* mutants, and two independently complemented *ccs2* transformants for each *ccs2* allele (*CCS2*) were analyzed for cytochrome *f* accumulation via heme stain and immunoblotting. Detection of the CF_1_ of the ATPase is shown as a loading control. Note that heme staining also reveals the presence of covalently attached heme *c*_i_ in holocytochrome *b*_6_. As evidenced from the heme stain, holocytochrome *b*_6_ accumulates to a lower level due to loss of cytochrome *f* assembly in the *ccs2* mutants. Different levels of holocytochrome *f* accumulation in the transformants might reflect differential expression of the introduced *CCS2* gene due to position effect of non-homologous integration of the construct in the chromosome. **(B)** Same as in **(A)**, except only one transformant was analyzed for cytochrome *c*_6_ accumulation by heme staining and immunoblotting. Ponceau staining is shown as a loading control. The white lines in **(A,B)** indicate assembly from independent immunoblots.

To test if the cytochrome *f* assembly was restored in the complemented transformants, we performed analyses of fluorescence rise and decay kinetics (**Figure 1B**). In such experiments, the emitted fluorescence of excited chlorophyll in photosystem II is taken as an indication of the functionality of the *b*_6_*f* complex, which receives electrons from photosystem II. As seen in **Figure 1B**, the rise and plateau curve for *ccs2* is characteristic of a specific block in electron transfer at the level of the cytochrome *b*_6_*f* complex because of its impaired assembly in the absence of membrane-bound holocytochrome *f*. When the energy absorbed by the chlorophyll cannot be utilized, in this case as a result of a block in photosynthetic transfer through cytochrome *b*_6_*f*, an increase in the chlorophyll fluorescence is observed. In wild-type and complemented strains, the decay phase corresponds to the re-oxidation of the quinone pool, the primary electron acceptor of the photosystem II, by the cytochrome *b*_6_*f* complex. This indicates holocytochrome *f* assembly and cytochrome *b*_6_*f* functionality is restored in the complemented strains. In accord with this result, we also showed that both holocytochrome *f* and cytochrome *c*_6_ accumulation is restored to wild-type levels in the complemented strains (**Figures 2A,B**).

### The *CCS2* Gene Encodes a Protein of the OPR Family

**FIGURE 3:**
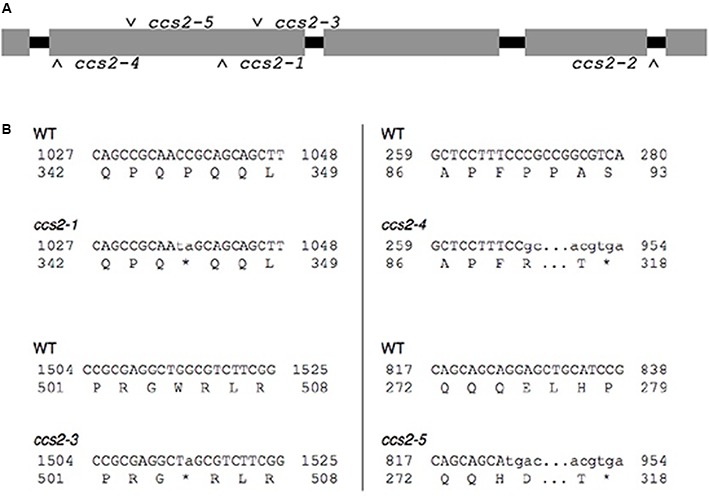
*Molecular identification of the ccs2-1* to *ccs2-5* mutations. **(A)** Schematic representation of the *CCS2* immature transcript. Introns are shown in black and exons in gray. The position of the molecular lesions in the *ccs2* alleles is indicated. **(B)** Identified mutations in the *ccs2* alleles. The top line corresponds to the nucleotide sequence of the *CCS2* ORF and numbers displayed to either side refer to position of the nucleotides within the ORF. The lower-case letters indicate the nucleotide sequence due to change(s) induced by the UV mutagenesis. The lower line shows the corresponding amino acid sequence and the change within the protein sequence resulting from the molecular lesion(s). The asterisk indicates a stop in the protein sequence. The *ccs2-1* allele is a CC to TA mutation at position 1036-1037 and the *ccs2-3* allele is a G to A change at position 1514. In the *ccs2-4*, deletion of a single C occurred at position 269. The *ccs2-5* mutation combines a G to T mutation at position 826 and deletion of a single G at position 829. The GenBank accession number for the *CCS2* nucleotide sequence is KC292647.

Because of the large size, low abundance, and high GC content (74%) of the *CCS2* mRNA, the corresponding full-length cDNA proved difficult to amplify. Instead, overlapping cDNA fragments, approximately 2 kb in length, were amplified and the full-length transcript was extrapolated by aligning the sequenced fragments with the *CCS2* genomic DNA. Sequence comparison of the assembled cDNA with the current gene model *Cre03.g213201* extended the 5′ end of exon 1 and identified four introns within the *CCS2* gene (**Figure 3A**). The *Chlamydomonas* transcriptome from the Joint Genome Institute, University of California (JGI-UCLA) and Genoscope^1^ corroborates this transcript sequence under the previous unique gene ID Cre19.g757200. We assigned the start codon in exon 1 based on the fact that this is the 5′ most ATG preceded by stop codons in all three reading frames.

Sequencing of the genomic DNA in the *ccs2-1* to *ccs2-5* mutants revealed molecular lesions in the *CCS2* coding sequence (**Figure 3A**). All lesions introduced nonsense (*ccs2-1* and *ccs2-3*) or frameshift (*ccs2-4* and *ccs2-5*) mutations, which result in truncations within the first 500 amino acids of the protein except for the *ccs2-2* allele (**Figure 3B**). In the *ccs2-2* mutant, a single guanine has been deleted from a stretch of 11 guanines in intron 4, 10 bp downstream of the 3′ end of exon 4. It is possible that this change impairs the splicing of intron 4 in the *CCS2* transcript. The identification of the molecular lesions confirms that the complementing sequence we isolated corresponds to the wild-type *CCS2* gene rather than an extragenic suppressor of the *ccs2* mutation.

**FIGURE 4:**
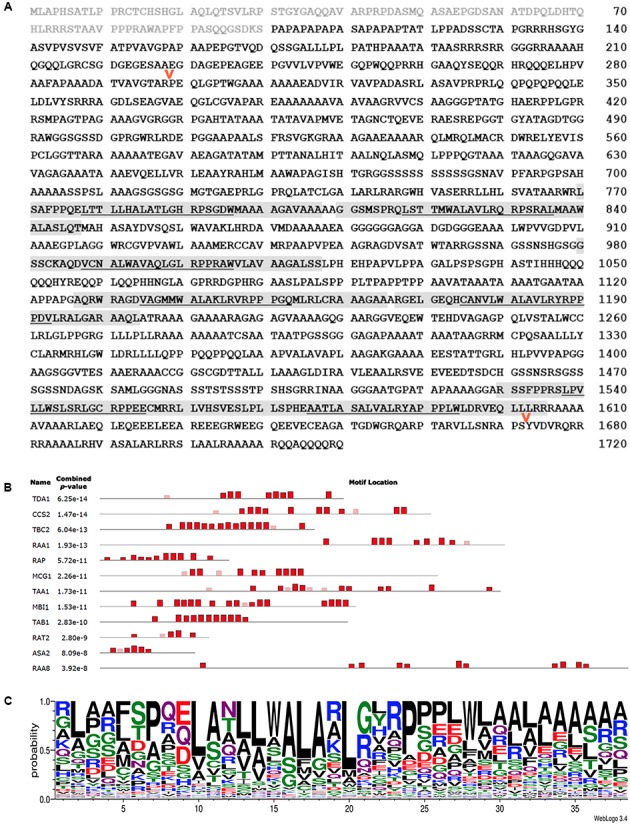
CCS2 is an OPR protein. **(A)** Amino acid sequence of the deduced CCS2 protein. Light gray text indicates the protein sequence that was fused to GFP for targeting experiments (**Figure 6**), light gray highlighting indicates OPRs recognized with *p*-values of at least 1e^-10^, as shown in **(B)**. The underlined portion indicates the location of the previously recognized “PPPEW” motif. The red arrows indicate the positions of internal HA tags that were introduced to test neutrality of the tag with respect to CCS2 function (see Materials and Methods). **(B)** MEME-generated distribution of OPR motifs within the *Chlamydomonas* OPR proteins that have been functionally characterized. Heights of blocks indicate relative proportion of the *p*-value 1e^-10^. Red boxes represent motifs found by the MEME program while faded boxes represent motifs found by other programs within the MEME Suite. Sequences of *Chlamydomonas* OPR proteins CCS2 (KC292647), ASA2 (EDP00850.1), TBC2 (CAD20887.1), TDA1 (CCA62914.20), RAA1 (CAE53330.1), TAB1 (ADY68544.1), RAT2 (EDP02536.1), TAA1 (Cre06.g262650), RAA8 (Cre10.g440000), MCG1 (Cre10.g429400), MBI1 (Cre06.g272450), and Arabidopsis RAP (OAP08625.1) were used (see Supplementary Figure S1). **(C)** WebLogo consensus sequence of OPRs identified by the MEME program depicted in red in **Figure 4B** (Bailey and Elkan, 1994; Crooks et al., 2004). Letter height indicates the probability of a particular amino acid (y-axis) at a given position within the 38 amino acid repeat (x-axis). OPR sequences used to calculate this consensus sequence are found in Supplementary Figure S2.

The *CCS2* gene encodes a 1,719 amino acid protein with a predicted molecular weight of 171,753 Da. The most striking feature of the CCS2 protein is the presence of several 38–40 amino acid repeats occurring between residues 720 and 1610 (**Figure 4AB**). Such motifs, named OPR for **o**ctatrico**p**eptide **r**epeats were first defined in TBC2 (translation factor for chloroplast *psbC* mRNA), a nuclear encoded protein required for the translation of *psbC* RNA in the chloroplast (Auchincloss et al., 2002). OPRs are also recognized in other factors controlling translation (TDA1, TAB1), maturation (RAT2, RAA1, RAA8, RAP), or stability (MCG1,MBI1) of chloroplast transcripts (Balczun et al., 2005; Merendino et al., 2006; Eberhard et al., 2011; Rahire et al., 2012; Kleinknecht et al., 2014; Marx et al., 2015; Wang et al., 2015).

The OPR family is further characterized by the presence of low complexity regions, which are regions containing little diversity in their amino acid composition (Balczun et al., 2005; Merendino et al., 2006; Eberhard et al., 2011; Rahire et al., 2012; Marx et al., 2015; Wang et al., 2015). Indeed, we noted the occurrence of several stretches of three or more alanine, glycine, serine, or glutamine repeats in CCS2 (**Figure 4A**). CCS2 has a high content of alanine (24.2%), glycine (10.7%), proline (8.7%), and leucine (8.7%), a feature shared by other OPR proteins (Auchincloss et al., 2002; Balczun et al., 2005; Merendino et al., 2006; Eberhard et al., 2011; Rahire et al., 2012; Marx et al., 2015; Wang et al., 2015). OPR motifs found in CCS2 are highlighted in **Figure 4B** and a consensus OPR motif from all OPR containing proteins that have been functionally identified to date, with the exception of NCC1 and NCC2 (Boulouis et al., 2015), can be seen in **Figure 4C**. The relative locations of the motifs in the proteins used to generate the consensus motif in **Figure 4C** can be seen in **Figure 4B**.

### Immunodetection of the CCS2 Protein

**FIGURE 5:**
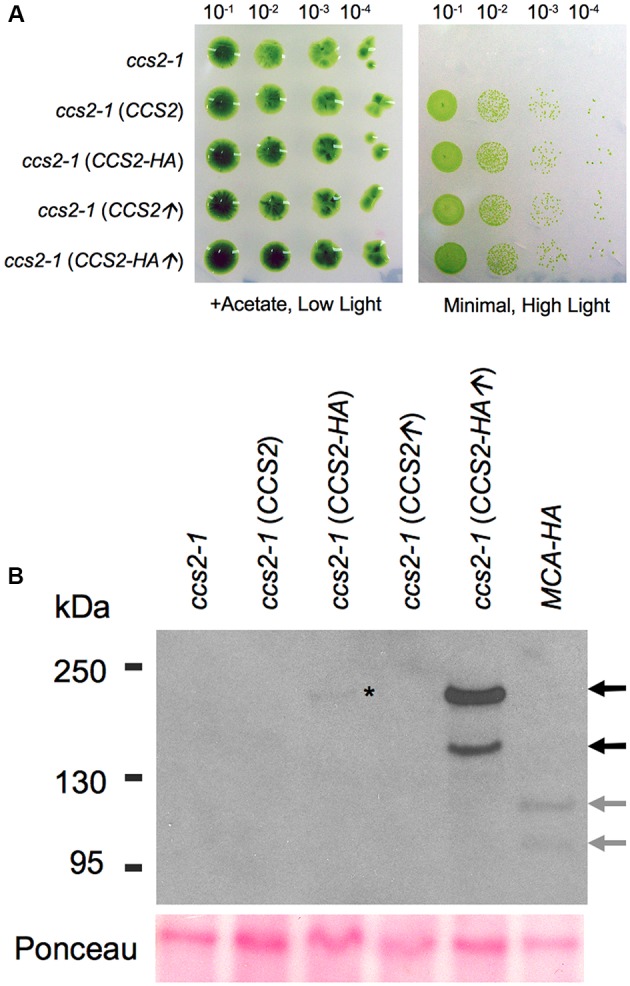
Immunodetection of CCS2-HA in whole cells. For **(A,B)**, a cell wall minus *ccs2-1* strain was transformed with plasmids carrying the 8.2 kb genomic fragment (*CCS2*), the 8.2 kb genomic fragment expressing HA tagged CCS2 (*CCS2-HA*), the *CCS2* coding sequence under the *PSAD* promoter (*CCS2*↑), and HA tagged *CCS2* coding sequence under the *PSAD* promoter (*CCS2-HA*↑), respectively. One representative transformant, selected on minimal medium, was used for the analysis. **(A)** Ten-fold dilution series of algal cells on acetate containing (0.6 μE/m^2^/s) or minimal media (50 μE/m^2^/s), incubated for 1 week at 25°C. **(B)** Anti-HA immunodetection using whole cell extracts. The HA-tagged MCA1 strain is used as a control (Raynaud et al., 2007). MCA1 is a plastid-localized PPR protein (PPR for pentatricopeptide repeat). Black arrows indicate the two CCS2 species, gray arrows indicate the two MCA1 species. The lower bands correspond to the expected molecular mass for CCS2 (∼176 kDa) and MCA1 (∼105 kDa). The asterisk indicates the CCS2 species of high molecular weight that is detected in the *ccs2* strain expressing CCS2-HA under its native promoter. Ponceau staining serves as a loading control.

In order to detect CCS2 and assess its subcellular localization, a series of constructs expressing HA tagged proteins were engineered. The tagged proteins were either expressed under the control of the native *CCS2* promoter or the *PSAD* promoter, which allows increased expression of *Chlamydomonas* genes (Fischer and Rochaix, 2001). We saw no difference in the ability of the two HA-tagged *CCS2* constructs to complement the *ccs2-1* mutation as compared to the WT gene (**Figure 5A**).

Immunoblotting analysis against whole cell extracts from transformants over-expressing CCS2-HA revealed that the protein occurs as two species, one of which appears to migrate at the expected size (≃176 kDa) (**Figure 5B**). A similar pattern was also seen in cells expressing HA-tagged MCA1, a PPR (PPR for **p**entatrico**p**eptide **r**epeat) protein with plastid localization (Raynaud et al., 2007; Boulouis et al., 2011). This suggests that the immunoreactive species with higher mobility may be a result of the extraction conditions and/or the denaturation step needed to immunodetect CCS2 (see Materials and Methods). Detection of CCS2-HA was only possible from freshly grown cultures when a cocktail of protease inhibitors was used immediately followed by denaturing at 70°C (instead of 100°C), an indication that the protein is likely highly sensitive to proteolysis. Interestingly, Rahire et al. (2012) noted that the OPR protein TDA1 was also very susceptible to proteolysis.

### The CCS2 Protein Localizes to the Plastid

**FIGURE 6:**
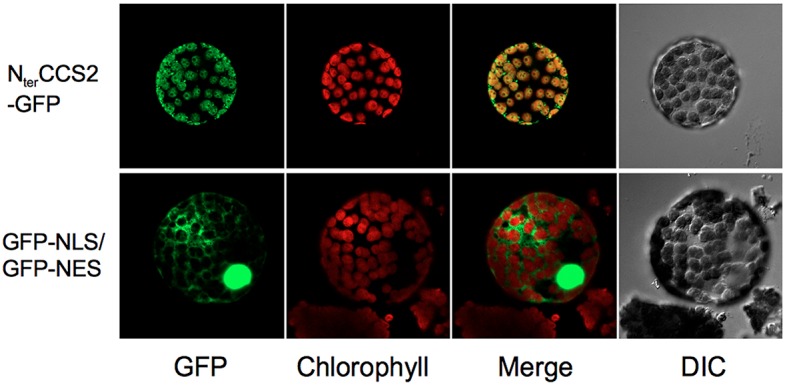
Localization of GFP to chloroplast via CCS2 N-terminal sequence. Top row (N_ter_CCS2-GFP): imaging of a protoplast from *Nicotiana benthamiana* leaves transformed with the pGWB5/ccs2target plasmid expressing the first 100 amino acids of CCS2 fused to GFP. Bottom row (GFP-NLS/GFP-NES): imaging of a protoplast from *N. benthamiana* leaves transformed with the construct expressing a NLS-GFP/NES-GFP fusion protein shuttling between the nucleus and the cytosol via a nuclear localization signal (NLS) and a nuclear export signal (NES). DIC, differential interference contrast microscopy.

A chloroplast targeting sequence is assigned by both ChloroP (Emanuelsson et al., 1999) and WoLF PSORT (Horton et al., 2007) at the N-terminus of CCS2, an indication that CCS2 might reside in the plastid. However, the extraction methods necessary for detection of CCS2-HA precluded the use of subcellular fractionation to determine protein localization. Hence, we tested the ability of the CCS2 N-terminus to direct GFP to the plastid in a heterologous system such as *N. benthamiana*. This is justified, as targeting sequences from *Chlamydomonas* nuclear-encoded proteins retain their function as transit peptides for import into chloroplasts in several species of land plants including *Nicotiana* (Nakazato et al., 2003; Falciatore et al., 2005; Levitan et al., 2005; Li et al., 2016; Yamaoka et al., 2016). To this end, we constructed CCS2-GFP, which encoded a protein consisting of the first 100 amino acids of CCS2, including the putative targeting sequence, translationally fused to the amino-terminus of GFP. This CCS2-GFP expressing construct was introduced into *N. benthamiana* leaves. Fluorescence microscopy shows clear overlay of chlorophyll auto-fluorescence, which is a feature of the chloroplast, and GFP fluorescence (**Figure 6**). These results indicate that the first 100 amino acids of CCS2 are sufficient to target GFP to the chloroplast of *N. benthamiana* and hence the CCS2 protein is likely localized to the plastid of *Chlamydomonas*.

## Discussion

### Identification of CCS2 as an OPR Protein Controlling Cytochrome *c* Assembly

Here, we have identified CCS2, a chloroplast localized cytochrome *c* assembly factor that, based on primary sequence similarity, appears to be unique to specific branches of the chlorophycean algae. This protein has been recognized as a member of the recently designated OPR family, which contains 43 members in *Chlamydomonas* (Eberhard et al., 2011). OPRs are defined by loosely conserved repeats of 38–40 amino acids (Auchincloss et al., 2002; Eberhard et al., 2011; Rahire et al., 2012).

Via bioinformatics analysis, OPR proteins have been assigned to the ‘α-solenoid’ superfamily, which contains proteins defined by similar tandem repeats, such as TPRs (tetratricopeptide repeat, 34 amino acids) and PPRs (pentatricopeptide repeat, 35 amino acids) (Eberhard et al., 2011; Rahire et al., 2012). Three dimensional structure analyses of TPRs (Das et al., 1998; D’Andrea and Regan, 2003; Zeytuni and Zarivach, 2012) and PPRs (Ringel et al., 2011; Nakamura et al., 2012; Shen et al., 2016) indicate that these motifs result in a series of anti-parallel α-helices. Protein predictions using I-TASSER (Zhang, 2008; Roy et al., 2010) suggest that the OPR proteins are organized in a similar manner (Auchincloss et al., 2002; Eberhard et al., 2011; Rahire et al., 2012). I-TASSER also predicts regions of anti-parallel α-helices in CCS2 (not shown). TPR proteins act as scaffolds to mediate protein–protein interactions and control a wide range of cellular functions such as the cell cycle, transcription in the nucleus, or protein import into mitochondria and peroxisomes (D’Andrea and Regan, 2003). On the other hand, all the PPR proteins described so far were shown to be control maturation, stability, or translation of organellar RNAs, presumably via direct interaction with their target transcript(s) (Barkan and Small, 2014; Manna, 2015).

The PPR family has expanded greatly in the plant lineage, with 450 representatives in *Arabidopsis* alone, while the typical non-plant, eukaryotic genome encodes for fewer than 40 representatives (Barkan and Small, 2014; Manna, 2015). Similar to the PPR protein family expansion seen in land plants, chlorophycean algae have an expansion in the number of OPR containing proteins (Eberhard et al., 2011; Rahire et al., 2012). The majority of functionally characterized OPR proteins are involved in translation, maturation, or stability of transcripts in the chloroplast of *Chlamydomonas*. For instance, TBC2, TDA1, and TAB1 are factors involved in the translation of the *psbC*, *atpA*, or *psaB* transcripts, respectively (Auchincloss et al., 2002; Eberhard et al., 2011; Rahire et al., 2012), while RAA1/RAA8 and RAT2 are involved in the maturation of *psaA* and *tscA* RNAs (Balczun et al., 2005; Merendino et al., 2006; Marx et al., 2015). In *Arabidopsis*, RAP is the sole OPR containing protein and loss of RAP function yields a defect in the maturation of the chloroplast 16S ribosomal RNA (Kleinknecht et al., 2014). Recent studies have uncovered the role of MCG1 and MBI1 in stabilizing the *petG* mRNA, encoding a small subunit of the cytochrome *b*_6_*f* complex and the *psbI* mRNA, coding for a small subunit of photosystem II, respectively (Wang et al., 2015).

If the role of most OPR containing proteins as transcript-interacting factors was inferred from the phenotypic analysis of loss-of-function mutations, the demonstration that OPR motifs interact directly with their relevant target RNAs was only provided for TAB1 (Rahire et al., 2012). A recent study revealed that gain of function mutations in the OPR motifs of NCC1 and NCC2 confer an ability to recognize chloroplast transcripts as novel targets of action (Boulouis et al., 2015). This suggests that specific amino acids within these OPR motifs must govern nucleotide recognition, but the basis of this specificity still remains to be deciphered.

The molecular mass range of the functionally identified OPR proteins extends from 45 to 269 kDa with an average length of 1428 amino acids and 5–17 OPR motifs. These repeats are found primarily in the central regions of proteins while the carboxyl- and amino-termini are typically characterized by stretches of single amino acid repeats (Auchincloss et al., 2002; Merendino et al., 2006; Rahire et al., 2012). The OPR motifs themselves can be highly divergent, and so the repeat count per protein fluctuates depending on how stringently one defines a single OPR motif (**Figure 4B**). This divergence is reflected in the fact that OPR repeats in CCS2 are characterized by a LWALAR consensus motif (**Figure 4A**) while repeats in TBC2, TDA1, and TAB1 contain a PPPEW sequence (Auchincloss et al., 2002; Eberhard et al., 2011; Rahire et al., 2012). While in some instances OPR motifs are serially repeated, the motifs can also be separated by gaps (**Figure 4B**), often by stretches of amino acids such as alanine. We noted the frequent occurrence of a poly-glutamine stretch upstream of the OPR motif in CCS2. Interestingly, glutamine-rich regions are known to form polar zippers involved in protein interaction but they have also been recognized as motifs for transcriptional activation (Courey and Tjian, 1988; Stott et al., 1995).

That CCS2 functions directly in the heme attachment reaction in the thylakoid lumen is very unlikely, considering its large size and the absence of a typical bipartite targeting sequence for luminal localization. Considering its inclusion in the OPR family, one possibility is that CCS2 controls the maturation, stability, or translation of a chloroplast transcript in the stroma involved in cytochrome *c* assembly. In the chloroplast genome, the *ccsA* gene encodes a heme delivery factor required for cytochrome *c* maturation (Xie and Merchant, 1996). While the level of *ccsA* transcript in the *ccs2* mutants was previously shown to be unaltered, indicating that CCS2 is not required to stabilize the *ccsA* mRNA (Xie et al., 1998), the hypothesis that CCS2 is involved in the translation of *ccsA* remains plausible. Unfortunately, this was not tested due to the lack of an anti-CcsA antibody. Another likely scenario is that CCS2 functions like a TPR containing protein and mediates the assembly of protein complexes containing other CCS factors. The OPR family also includes ASA2 (ATP Synthase Associated protein), a subunit of the unusual mitochondrial ATP synthase, found only in chlorophycean algae (van Lis et al., 2007; Cano-Estrada et al., 2010) (**Figure 4B**). While a 200 kDa CCS complex containing *ccsA* (40 kDa) and Ccs1 (60 kDa) has been identified at the thylakoid membrane (Hamel et al., 2003), it is unlikely that the 170 kDa CCS2 is a component of this complex because of its large size. However, it is conceivable that CCS2 could stabilize the CCS complex via protein-protein interactions or facilitate the recruitment of the components of this complex on the stromal side of the thylakoid membrane.

## Author Contributions

SC executed the experiments, interpreted the data and wrote the manuscript; PH designed the experiments, interpreted the data and wrote the manuscript; IL executed experiments.

## Conflict of Interest Statement

The authors declare that the research was conducted in the absence of any commercial or financial relationships that could be construed as a potential conflict of interest.

## Footnotes

1 http://genomes.mcdb.ucla.edu/Cre454/
