# Supplementary material for: CCS2, an Octatricopeptide-Repeat Protein, Is Required for Plastid Cytochrome c Assembly in the Green Alga Chlamydomonas reinhardtii
Source: Front Plant Sci. 2017 Aug 3;8:1306. doi: 10.3389/fpls.2017.01306 (PMC5541062; doi:10.3389/fpls.2017.01306)
Supplement: Supplementary file 1 [file Data_Sheet_1.doc]

**Table S1. Primers used within the context of this study.**

| **Primer Name** | **Sequence** |
| --- | --- |
| CCS2.69 | TACTGCTACAACCGGGCGGCTTC |
| CCS2.66STP | CAGCTACAGCATTGCTATTGCTAC |
| CCS2.19 | ACTGCGCCAACGTGCT |
| CCS2.54 | GCTATGACTGGGCGTGCTAC |
| CCS2.21 | GCCGGCAGTAGCAACAGT |
| CCS2.18 | GTCCACTCCTGCTCCACAC |
| CCS2.79 | GCACCTGTCGGTGGCGAC |
| CCS2.02 | CGTTGGTGGTGCAGGTGTAA |
| CCS2.27 | ATGCCGACTACAGCCAATG |
| CCS2.28 | AGCGTCGCCAGCGCATGAAGC |
| CCS2.51 | CAACCGCAGCAGCTTGAGTT |
| CCS2.26 | CGGCCTGTGTGTGAGATG |
| CCS2.81ATG | ATGCTCGCTCCACACAGCGCGAC |
| CCS2.70 | GGCACTGCAAACCACCCGACCTG |
| CCS2.t1 | CACCATGCTTGCTCCTCATTCTGC |
| CCS2.t2 | AGACTTATCAGATCCTTGTTG |
| 10aa-F | CACCATGGCAGTACTTGGAGGTCTTGCTGCACTT |
| 10aa-R | AAGTGCAGCAAGACCTCCAAGTACTGCCATGGTG |
| CCS2-HA_BsiWI-F | CGCCTCTGGCGTACATCAACGTTACCCATACGATGTTCCAGATTAC |
| CCS2-HA_BsiWI-R | GAGCAACAGGGCGCCGTACGGGCGTAATCTGGAACATCGTATGGG |
| CCS2_HA_BspEI-F | GTTGCTGTCGGCACAGCACGTTACCCATACGATGTTCCAGATTAC |
| CCS2_HA_BspEI-R | GTCGGTCCGAGCTGCTCCGGAGCGTAATCTGGAACATCGTATGGG |
| CCS2-PshAI.R | GAACGAGGTGCTTGAGGGACAC |
| CCS2-BsiWI.F | TGTTGAGCAACAGGGCGCCGTAC |
| CCS2-XcmI.F | GAGGCGGCGGGCACCATCCT |
| CCS2-XcmI.R | CAGTCAGTCGTGTGCCAGATG |
| 8.PROM-f | ATCCTAGATCTATTAATTAATGCTCGAGCACACACCTGCC |
| 8.PROM-r | GTGTGGAGCGAGCATCCTCGATGGGCTTGTTGTGAGTAGC |
| 8.3xHA-f | GCAGCAGCAGCAGCGGCAGTGGGAATTCTACCCCTACGAC |
| 8.3xHA-r | CACTAGTAATTTAAATTCTAGCTAGCGGCGTAGTCGGGCAC |
| SG1 | CCACTATCGACTACGCGATCATGG |
| PH3 | GGAGTCCCGGGATGGATTAAGG |
| CCS2.69 | TACTGCTACAACCGGGCGGCTTC |

**Figure S1**. OPR protein sequences used for the creation of Figure 4B.

>TDA1 (CCA62914.20)

MLSRASASLFAACGPQNADNCLTPSPSSSLLQPARSALASRLNSASGRRSPAFFVTTGLRSRVILASCSLRQPGPPEDANSDSSPHPPAAQPSTPPDVPGGTARARSTHLGGTGLGDGPLSGPDAVPAPHQSSSNSGIGGSGGSSGSSGRPVDNSSTGSSGSAAGSEASSSTPTSASPDSGPQTMSSTGSGAEGSSVAGGSPHQAVPPAAAAGRPYLNGHAGPSAMAPGGEADGAAADDRPGVGPSSLPPLRLDFLDLWAKMQMPPQDGPAFGGSGWQLGQPPASTPSPSSASSPSARSGPSPAGGGADKTAGRYPRSSGGGASGGRRRRESATRSRDERARQLVLAAKTLVQLRLVVERHLPDMGPGTAALALSRLAGLAAATQRLRRAQQEPQLPQTQSQQQPLPSGVVASAPVQEQQQADGVGPLADPSAAAGEAPSASMPGAAPSAPTASAAVQLPPGTLQVELPATASTSAATGAAIGTVDEDDDSDMDNVEGGAVGVKPGGRAAADGGARVPRSSLRLMRRMLYHIAASVAPDLYASPPQEAAAATASASIASTSTRDRTATSSTSTSTSSYHVGRGRFSPPEHRAKLAGSVLAAMARAGLRPDAEDSRALSALLEASRPDLFLATPAQLCRLASSLPLLRLAPSSRWLDHFAAVSRSKMYAASPLQLAAYVWALARLGYHPGDLWLYSLTQQLSRDGRLAAASAGQLCEVLWALHRLGFSPDPDWRAQAAEAVAFVAAREVRSVGGGGGGASRSGGAPADMEAEEESIQDGSWSAVSGDSGSRGGPMASGTRPDTHGATGAATQWSAAGSAGGLSGGFGGGNTAAEMCSLLLWAVRTGTAPASAAMEACLDRLQPHLRGLRRVHLLRLLLALAESGHRPREELMARVLGCLQPKLASMSATHLTQALVSISQLRFVPPQSWLMAFLCASRAQLRHYTPAHVTATYQVFAGWQLRPPRSYLEALHTYMDGLLPGFNAKGLATVLQSMASLGIKPQRRWLLRALEALADCGARGIAAGSGAVLAEQFRSGQQPVQQVAGAGVAAAGAAPLGEEVAATAPRAGLSAAQQQHQLRQQQRAALATSLVIALCSLRGVVQLEAQTQAQQEASDEAKQAPTATAQESLDASEVAASVAAAANTDGPTAAAAPAAASLLPYELWPGVKAVADVSRELLDAMSGPQLARLVSALARVNFYPGREFLAAHSRATARAGAQLLPEDKEAVAGCYSRLAALAPLMASEARRQAELRQARTAVLVADPWAGA

>CCS2 (KC292647)

MLAPHSATLPPRCTCHSHGLAQLQTSVLRPSTGYGAQQAVARPRPDASMQASAEPGDSANATDPQLDHTQHLRRRSTAAVPPPRAWAPFPPASQQGSDKSPAPAPAPAPASAPAPAPTATLPPADSSCTAPGRRRHSGYGASVPVSVSVFATPVAVGPAPAAPEPGTVQDQSSGALLLPLPATHPAAATATAASRRRSRRGGGRRAAAAHQGQQLGRCSGDGEGESAAEGDAGEPEAGEEPGVVLVPVWEGQPWQQPRRHGAAQYSEQQRHRQQQELHPVAAFAPAAADATVAVGTARPEQLGPTWGAAAAAAAEADVIRVAVPADASRLASAVPRPRLQQPQPQPQQLELDLVYSRRRAGDLSEAGVAEQGLCGVAPAREAAAAAAAVAAVAAGRVVCSAAGGGPTATGHAERPPLGPRRSMPAGTPAGAAAGVGRGGRPGAHTATAAATATAVAPMVETAGNCTQEVERAESREPGGTGYATAGDTGGRAWGGSGSSDGPRGWRLRDEPGGAAPAALSFRSVGKGRAAAGAAEAAAARQLMRQLMACRDWRELYEVISPCLGGTTARAAAAAATEGAVAEAGATATAMPTTANALHITAALNQLASMQLPPPQGTAAATAAAGQGAVAVAGAGAAATAAAEVQELLVRLEAAYRAHLMAAWAPAGISHTGRGGSSSSSSSSSSGSNAVPFARPGPSAHAAAAASSPSLAAAGSGSGSGMGTGAEPRLGPRQLATCLGALARLRARGWHVASERRLLHLSVATAARWRLSAFPPQELTTLLHALATLGHRPSGDWMAAAAGAVAAAAAGGSMSPRQLSTTMWALAVLRQRPSRALMAAWALASLQTMAHASAYDVSQSLWAVAKLHRDAVMDAAAAAEAGGGGGGAGGADGDGGGEAAALWPVVGDPVLAAAEGPLAGGWRCGVPVAWLAAAMERCCAVMRPAAPVPEAAGRAGDVSATWTARRGSSNAGSSNSHGSGGSSCKAQDVCNALWAVAQLGLRPPRAWVLAVAAGALSSLPHEHPAPVLPPAGALPSPSGPHASTIHHHQQQQQQHYREQQPLQQPHHNGLAGPRRDGPHRGAASLPALSPPPLTPAPPTPPAAVATAAATAAAATGAATAAAPPAPGAQRWRAGDVAGMMWALAKLRVRPPPGQMLRLCRAAAGAAARGELGEQHCANVLWALAVLRYRPPPDVLRALGARAAQLATRAAAGAAAARAGAGAGVAAAAGQGAARGGVEQEWTEHDVAGAGPQLVSTALWCCLRLGLPPGRGLLLPLLRAAAAAAAATCSAATAATPGSSGGGAGAPAAAATAAATAAGRRMCPQSAALLLYCLARMRHLGWLDRLLLLQPPPQQPPQQLAAAPVALAVAPLAAGAKGAAAAEESTATTGRLHLPVVPAPGGAAGSGGVTESAAERAAACCGGSCGDTTALLLAAAGLDIRAVLEAALRSVEEVEEDTSDCHGSSNSRSGSSSGSSNDAGSKSAMLGGGNASSSTSTSSSTPSHSGRRINAAGGGAATGPATAPAAAAGGARSSFPPRSLPVLLWSLSRLGCRPPEECMRRLLVHSVESLPLLSPHEAATLASALVALRYAPPPLWLDRVEQLLLRRRAAAAAVAAARLAEQLEQEEELEEAREEEGRWEEGQEEVECEAGATGDWGRQARPTARVLLSNRAPSYVDVRQRRRRAAAALRHVASALARLRRSLAALRAAAAARQQAQQQQRQ

>TBC2 (CAD20887.1)

MLPLEHKASGRVQATGRGVRASVELSSVLPQQRAAQLQHQKCNTGARLGRDPRRGVDAERTLVCTAATTASVPSTSGASPSGSQLSSKALRPRRFSAPVIARLLRSTTTVQELADLVQQQSLYMDSSHVGIAMLHLALLVSRAEQQAAAQLQLQLAAKQAATRRAGSGASTSGRARGWGSGPGRNGSGSSSVSVNGSGSSSNGSSSSSSSLAMGMQLSMASIGDDVVSGVNAGPVPSGGADALLDLEMSSILDDDDGAGARQLQQMSDDLAAGLEAAATTTAAPEAGVAAAGGTGAGAAADAAASSSAPSLVAAAAAAAAAAASPASSPDVARTLRTLLSRAFSLGLDSLSGPQLAAVFTGLAVLRRPRQQQQQQQQAGAAGAGANAGAGGVGGVGVSAGDRLVAEQLLAAMGPKLYECRPQDLANTLASVALLGLPPDADLRTSFYAAVRQQQRRFGPRELATTLWAYGAMGTYVQEDAVQLVLELSRARLTSFSPLQLAKAVQGLAALRYRPSPEWVEAYCSVLRPALRRMSSRELCAVLLALASLQVGLDGGTRAALLVHTFSGPLPGMAPGEVALSLWALGRLSAVDMDLPALIDLDMSGRVLDLTSRLLAAGGFSGGELQQLLEGLTRLALQPPLEWMQAFVAALQPQLDKLDAQQLAGVLNSLAAQQYRPQPQMQEVVLAATQANMKQLLADTTCSAALLTALRRLNIEPPPGWVGALLEESRSALKNRCTDLHLANLAGSLAAWGVRPDGRWAARLMWRSQVLMNEDRMSPRALVALLQAMVSLGLSPNPVWTQLCLQAAVRRASQPAFEPHHYGTLMASLHALGIQPPQEWLTRMLLSTYRCWDRFSVTHWSSLLPALVLLKARPPREWLRRFEATSAARLADCSALQLLTLAVSLAQLHQLHAAGAVADTPLLLPGAAAAAAAAAPAGASSAAAAGDSPAALSAVPAAAGDGALVPSFMSIDDDGTAAVAAAATALAAAEPAAHAATSTTTATAVAHPQPQLLPQAQALPQPGPEWQAAWWAASTRLLLRVRYAPSELVLTAGWLGSLGLRPPPEWLQACAEVAARYSKVMDAAERQQLAAAVAPLALEAVAPPSAPPAGAASTAH

>RAA1 (CAE53330.1)

MRRHPTCGIATPQAYGVAWPRLPPVAPRPASGQLPPRCRRAVLCAARRPAGGTRAASGADAGGSAGEGEGATAAADSHGTRSRPTGLEGRSASGSAVPAGIGGGAGSDSDGDAESAQRSGPRRRGRPPGMRQTMAVAPPTPNAGSAPATALAASSAVQQTVGQAANSPGSGSDGEGSGLATRRRGRPRRSTADASGGGCSIGGSTRAVTSAADYEAASAAAAPMPGMPLASGRAEPQPQPQPQSQPQLQQQTEAGGSTATAPGGAGAGSSGSGSTQRDGRSGAGGGAGGPLSRLLRSQRSRAMGGVGGAAPNATMPGGAAPPGDDSLQFASPMSSFEPAPERSSESDTEGGAVGRSGGATSRRQTPPPPDPQPPHQQAPPQQQQQPPDAAGAASAGSMASTDWMLGSMLEDGEAGSGLAFGSPGGASSGSDQDFIITADLDVVGSTAPPLNLDLSDLDWGDDTGAGGGWGWGDASTGGSAGSAMGALDGVGVGLGAATSGIIGIWGGDASASAAVAGDASALGQTPGSAAAALDSWPGVLQDDEAPQPQQPQQPQPQQPQQAPQAPQAPQALQTPQALQAPQGRPPVLLKGPAAESGPSRPVPAGDGGGAEATQSGSRMDSHPHPGLDSDFDLVSSYDAEMSYPDAVDCLLPEGVAAAAAAAGAGAPGGGGWSEGVLDSMADAGLPDLSYSGSGGGSGSGGGGGSSEGGKDSAHEARLPVAVAGAGRAPVASSSTPIAAAPAVPATAPAAAVPAPAAAKAAAGAAKAATGAAKGGAQAASGSSGSSSGSSIDGSSRTAAAFAELLAGGSSAHGAGRADPLVQRLARVLSYVESELLGAPEEAAAAAADAAAELPGVSLPPLPRPLVPAAAQPLELFAEPPATARLLAAAEAAEAGGLAAPPRQVEAPPSADEWALLGGAAPASAASAASAASAASAAGTSRAGPGRYGGGAGYEDDTDGGSSWEREREREGDGEADFERRLEAGPAYVAYLRAVVLGGLGGDSGGGSGGGGGGGRGGGGPVQAGAVQAALLEQVEAAGDWLQLCWLLEAAAAQGVALGPRAAAAAFKQAAGLVAPQGALPRSLAAATTTATTSGLEAAAYRQLCDRLAAAGVAALGTSVSAAASTHTAARAAGGRASTSRRGVPPGITAGGRGGGGGGAGSHVSAASGSDVAQISYGMGALQITCPQLYGAVLHVSASQLAALTARRRAAASLTAAAAAALRRSAAAVPTDSAGRGGGGGGDGVRRVSDREIMMAAAAASSAVQPNHASELPPQQLQQQQQQQATSGRGPLSGSAATAKSALTAAAGGVWTAADLAALAWGVANAYAGSAAAVAASAAATADSCATASKAQRQQRQQRQPQQPPRPPKHRQLQGTQQPQPQPPLPLPVPGGDWLRSLVGASYELMATEGGGGGGGGGVGPRQLWGLAWSFARLGYAPSQEWMLALLSRAEACLSQFDTEGLCRLLWALAAMDYVPERLWLRAVAGQLQARARDFTPDQVVTLHCALARLGYAPRPEVCVALHAAAARLMPLMTGPQLAALAHAAASFARWRPGPGFLVAAASATGGAAAPVPSASSPAAAVQMPAGLLMALATRVLVGMRQATTEAAAVASSPAGGEAGAWAQPQPQPTALAAAAVDGSDLSMALWALALLRRQQQQQQQQPAADVLQLMPEWLAAWWAAAAEPAVAATFDATCVSQSLWALAELRETPGLPHSGAAAAAAAAASGGAASSTYADAARDAGQQAAAQAAAGAVAALLAALVPQLGQAATADLSTTIAALADLQYRPSDQWMALFTAEARRRLGTATATATATGTATTAATATNEDHGLIAYGLAVLGWPLSEAWVQELAAGGYRAMAGASGEGLALLLWGLSARGWSTASGRFWDTVFRESGSKWDSCGPRGAVLLYCAVADMMPPGQEPPIPWQRQLVKALRLRVRPRPRTALLLPAALRTAAGGCLGPLTRAERQQLWQAGAAAAAARAAGGGAGGIGLVLGAEAYTAVPGSGLAGAVWWSALPQPHPRPPSLPGVKGWAWPQGGPQGSGADEVQDPEEVGVLASRVLLPCELCEPEEHPALAAAVAEGRSWWAHDVAAELARRWGLVRWS

>RAP (OAP08625.1)

MECVVPFRRCFCLNPPETRHRIVNHNHRNLHISLSSSSFASGILPLSNKKYRFVGPLAQRSSLHRRTDSLKHLPFSVNASVIGNSEEEVEEEDDDGDWEAEFLGEIDPLDIQPPKKRKKQKNSKALEDTEGMDWCVRARKIALKSIEARGLSSRMAEVMPLKKKKKKKSKKVIVKKDKVKSKSIPEDDFDTEDEDLDFEDGFVEDKMGDLRKRVSSLAGGMFEEKKEKMKEQLAQRLSQFSGPSDRMKEINLNKAIIEAQTAEEVLEVTAETIMAVAKGLSPSPLSPLNIATALHRIAKNMEKVSMMRTRRLAFARQREMSMLVALAMTCLPECSAQGISNISWALSKIGGELLYLTEMDRVAEVATSKVGEFNSQNVANIAGAFASMRHSAPELFAELSKRASTIINTFKGQEIAQLLWSFASLYEPADPLLESLDSAFKSSDQFKCYLTKEITNSDEVVDAEVSDDVSRSPALSFNRDQLGNIAWSYAVLGQVERPFFANIWNTLTTLEEQRLSEQYREDVMFASQVYLVNQCLKLECPHLQLSLCQELEEKISRAGKTKRFNQKITSSFQKEVGRLLISTGLDWAKEHDVDGYTVDVALVEKKVALEIDGPTHFSRNSGLPLGHTMLKRRYVAAAGWKVVSLSLQEWEEHEGSHEQLEYLREILTGCI

>MCG1 (Cre10.g429400)

MRTTGWATTAGGHPFAARGKLRCDLVTARAAAPHTGNTNRSEARSSLQIHGPPAAAESALVSVTPDALVRPLKVNGHATHGSSEPGSGRSSTSATLAGNTDACGLLTGARPVPADAAKSVHSLQIAPEHSLVPAANGRSHPPAATSRDAPPLVRGAPLRGAPARETGGRGQDEDAAAGAPAATASWEYARTAEQQHVPPSPASTSSASGSSASLNGWPGAHDSGGGGSGGGRGVAYLGRGSSRVAHSYRPQEELEQPQQRQQHTQATSNYSPSSRGAGGGSRQPGMSYFTGGASARGSTGPANTGGATSAGASAGVWPSGADLVNAGLLAKIDAATNWYELRAVLASSGGGSGSTSTSHSRSPRNAGGGAASATAAAASSPSAAATALAVLRRLAAVTRYDMRPAECAALGAFLERWLLECALPALPAMRAADLAAAMHAVAKLARALPAPPPAWTAGWFAAAAPHLRAAAFRPKDISLSLWALSKLAARPPAGGLQLLLAAAEPHLHRFNAQDLSLVALALAALRGRDGGVSSGSGSGEGSTDGEGRGSTGGEGEGLQQLLPSAEWRQLYLARVGEVVRQEAVGALGSSTSQLGGGTSSYSGGRGGDCGPQALCNLLHGLVRSGLLSSSVPAPPDADRALPPAGLAGTDGRGPAAGPSWLDEEGGEELAMAVVASATLSPVSAAVLAPSPSSPLVAAAAVDARWLLEDLCTALYSVLPQCTPQGLANALSALAAAGHVPDAGWLERFYIESAARMDTGSSSGSSGSSSGSGREGADGADSSISGGNGSSSRWCNADDLAHMAAAAAELRLAPPRWWAARLYGAMERRLTHGGGGCSARQLSQMLHGAAQLHRSYGQLPLAPVASNTKSGAGVDALAPHAAAPVVAPAPPCTVAAAAAARPPPSLLAAWHRAAAAALPGFNAVDAAHSLWALAALGERPPPDWLQRLLVGVRGALVAAPPSELAVLLWSLSELRFRPTWSWIADCVEASAPGLPRMEGQDFAMLLSGLVRLGGRPTPEWSARLMAALAPRLRRLRLRSLSQCVWALYRLRVQPPPEVLTQLAAELRRRWLQQAAAASASASASASAATSAAAGEEQREQLRHSVMLLWVISIWLGSEGRKHRPAVAAALAAGGRNGAGRGPGGRLTGAPRLRLRRPVNGNTGAAGLSSVEPRSPDVAIKQQPHSAVAAGPQTHRPPAPACRLSSRMLRRRRAARPGHLAAALGGDAAAAAALRRSLASQLLPAALAATEPLLAARRATAQDAALLAAVVRRMPRQVRRAAATAAPAWPQALLDATEPLLPSMTHAGLLQTLAAAKLLLRGSGKEQGAGAVVDGRWVAAAERAVAVQLGAASPLLPCAARVTLLRRLTALGASGSAARLNGASMPCQPAASNGSSSSSSSGSSCGCGTATTPAGGIRTVTAAGSSRVLLDAVAAMRRTGTVAEPLVALQAALLRARVRLRKLRAALRGAHAAAAARRARGPPPAASAALAAAAQDVAAATELAVAAAAGAQSACRPKTLAAAQWRVQAAVVAATYALLPPRGSGSGGLADEDDGEEEDVAAPAVRVNGGRQPLPPLSVEQRRWIQARAERTVAAQLLAAAPPEHVAQLAALAAVPLAAQAGRAAATGASQQAGGPSHRRRQQEQEEAARSLLWLAAACSARWRRMLRPHRALAARSWRAALASLELSSPALAQQLAAVLPAVVSGGARPGGHGAGGAGDMTGESDCKAAARAAVCAELAAASGVVLEAATSAAANGR

>TAA1 (Cre06.g262650)

MQSHCAHYKAGAAARREKSSREISSSAPTRPLHCRSSQPSRAITFAINLRADGSYDAGLTRPQAARQRARRRTGPPSTATVSRWSASDSDASRFDWLDGAKQQQTQVLVDALFAAESVPPPRLPVLLALLEGRPDLEQALVEAGLPAAAAAVLRSDLLQRLLPGGLLLETLMGLIEDRATELPAEQLLMACRLVAAWHPPNGCEALGPASASLATAIADAVLAGPDSTGEADKADSRGSGGSSADFAATAGMLADALTLLQPWLAWRASGSGSAAAAAGTSAASAAALGLLDAGVAAAEEAAEAADALAAARGAAEALLRRPEVAAAADTGVLLAVTAAAAAYDVEVPRDLARTAVSLASTASSSSPTASAGSSSTTSSGAGSSSATTAAELAAQLVAASVAGAAGGAAGHGRGRGRNQLSGEELESVVWARLASEVGAAEHAAFLRELLGRVRQMAPGSGRLQLQPEEQQHEQQPGSKAGEAGPADEQQAARRALVVALGSAAGGLGPDALALAAECAAAVFPGDTIGDGADALASTAAAELLGCAADASAEPAAACTADQVCRLAAAVLALRARVGASVNETAVARVLAAAEAVQLQSASTDAVAALVAAAAVPGGGLALPPPLVDALLVQLTAAAATAAPVEDVAVDGSAGTAKSGRKQEAAPAAAAVAQLTPQQAAALLEVAVAAAEEAAQAAAAAVPSSSTAAEPATPAPIPPQRAAVLCAAIDVAMRLLTPSVRRVDSVSDITRLLVLAHRCQRAGLRPREQRGLLWAAHERLRVLGLSMSPAEAVGVLRACAALKWAPSVLFSELLLPLLRQLQASAAAAAAAAGGPSPASGAGSTSGADDTSALGAWGEAGSGGAASRPWTLREVRSALALLAAVGYDGPMAASLVKLGVGELLRAHHVAATAASRRSSSEAGAEGEGALLGAEDMTQLLWVCVALRYRGGAVLRPLLQLLLLVPAPQVSVRAAAQAVWAAARLGVVGERLVRWALAACQGQGKLAAAPPQSLANLCWGLGKLGVKPPRAFVTAMAVASLGQLPHFTPQELATTAFVLATWGGRLGAAASGLVRHVVATRSHFDGPALCVAAWAVQRLAAAPPADATADAAASADVTPGLDAASLGLLEQRLLEAVQEAAHERRGRPGTGDFLQSLPGVADHHLLRFFSAASAAGYRPAALLAAYCDALLPRLQRFNGCSPAAAAGAARRARLLWSAARVLQTFRVTAAERPELLAALEHAAEQCQAILGPQALAGVLSAMSDLGHYPAGWAARGLLRCVRLGLVEADATEAAAIVAALAAWGERLEEGEAAAAAAAAAAAAGAEDGRQGNGLAAAGGEEAGNPSRAAKAAKPAPSGKQAVSRRLDLLRQVAVARLTELCVPPPVVAAAGPGAAAAAAAHGQALPSAAGAAAAAPSPAEASGAGLTPAAAATAAAAAEAPSAGGAIAQPSIEPEVALSLLRSLARLRWHSDPLEGALVRAAAALTADPAGRRRVPALTTLLWAMASLRQDVPELLDDLQAALMGLPRNTRSLAEEVALLESGRLAGALGADSQRPPAPPSVPPVQPVQPVQQHSQAAAEPTPVVEASAQPTVVLAAAQAAAQPRPSPAASPSAPSTPSNSTTASAAASNNHNHNSTNGNGLLARPNKPAVPTPATADTVSPTASLSSLSSLSSMDGGAGGGFDTAAATAAAAAALNQRLEQQLVGAAGRGRLASEPWAPVDVFKALWACAKMNRHPGPQILAAAERSWVLHTTDGAAGGAEGRTLPPLHTVTGLLWSLSVFRHHNSAFAQQLAAQLAARLGVLAAAAAADGGEADGAAAAAAALEKQAPQLAACLLAAAADRTDSPLNAALAPEARGRLLNVWRARQAERVARPPGRYQTDLVSVLRKMGYTAAANVATPDGVAVADVAVAVTPNAGLRAASTASASPGTSSTSSMDSGDGAAAATAATADSGLGSGAAAAAAAAPAPAAPRLLALELVGRHNSAANSPRIMGEAVIKYRLLQAHGYLVVPVSCYEWDRISHQDVWTKMVYLQAKIDRRTGTGLAASSVSAAAAAAAAAPAAGAEGQTQTQAVSASSGLSRSQPQ

>MBI1 (Cre06.g272450)

MWKEGLSSSSLRGCARNAIYARAQLAKPKLDGRSAEPASAHANHSWPPSAGIQVSLQHLPARTQEPQHAIAVERASVASADPTLAAQPLNEEFERPAGSRSSADCAPSTSARGYAGGSKQPRGSFRRGVDSAGGNSRDAAALTRRITHSKTTSELHDVVTRHKSQFNSIHTAAAIVKLAKLTAGEPEQPHHQQQHQRHQRHHRQAQAGLHEVVGNGHLQAAAPVAGAAADGQLGSSSADASGADPAAFGTPGSRNQGQLFRSRHTPSRGAAAAAAADDARLRESLLEELSEAFLAHAQQQQYPSARQFANVVWALGSMRIRSPRQRPDTPPASSPSASSLEPQPQPGTSTTLQAIAGEPQPQPSSMLQLGPLLSVTAAQLLSGNGSRLTSALPQELSNLALGLAKLGYREVPLWAAIIAAGKARLPAFKPQELHNLAWAVAAASQDRSMISAAVQAALPQLGAFTPSGLSNLLWACATAQCHVEELFDGAAAALLRVPPAQLNSQDVANTAWAFAKVQHPHPGLMRHLGGLVLRAATGRDAVAGSGATDAEAGTAAGAGAMQGPEAGTGRGLRGVATQELVNVLWAFASMPPQGLGLPDAAAGGVPAQGRGSTWGGVNASSSSGASTSSSNTSGGGGGGSTAGLTTQLLAALLPEVVRRRDLTPQGASNALWAAGRLQPCPVPPDALADALRAASTRAASMSDQELANALWAAGELRGAGHYVPPAAVAPLFAAACCPSRLEATPAAGVAQLVSAAVKLRLVGSQHMDALAKRVMRGLGSLGPQELCVLAAAVAEAVHVAAYCNPILLNGLANAAVAQVDRLDPQGLSTLLWAFARAGKHYHGPLTTTICRVAAPRLREFSDLELSNLVWALAVLKCQDRQLLVRAARVLVGRVRLRRQRQQAAAAGAAGLAAMQQQRRYWSGQMADGSMPPQQMQTQQQQRAEAEAQRRLGLGLRQGPGQDAGGPVRIRLDAPAAASASGWRRAGSDGGVNAAAQPHPPESPSPAAAHLGSNGNGTAPAASGSAPIGSAFVSTATAGSATSSMDEGSLPDDRPLLRRLPSLHHHDETHVMLQARRPHPRDASAVAAAAAAAGAVAAASGSAAGGGRGLQSCDGSDVPAAVPTVRRRRLTDSLSDGEDPEGGSAGLGAGGAAPTAQGHAKSMAKLLWGFAKCNLYNQALYRLLVQELRPLMHLLTPHEVVQVLWSVAYHSHSCPELLDAAAPAIASRLGRFCPWDASVVAWAYAKLDHPHRDLFESLQHHALRYGSRYKEPCLLRLVWACAQLQLHVREPLLAQLHALRTGRSRAASSYDGADGEPPPPSDAGRMEPEWW

>TAB1 (ADY68544.1)

MMPSLKNLGGGFGRVSVGTTPFRKCARAAGRRAAPGLCSAHSAGESPLLSNTELERRQTGAQRQVVPSRRQSGSGGPGAPPLAPSQLTAAIHACTSTAQLLALYRQHGKRFTHVQASAALRQLVNVAPLPAPESEEDAQGGASSSSNSARSPEALAARQVTGRLTVRQGEGEVVAEQRRNSNYEASTSAAAPAPAQPPSKGKGKRPLLQDPEPTTTADLALTEQPSDASTIGTLAPQPDVAAALPAAVPQPQPSRRQITSMAAGLAAVLSHSCGVMDGRGVATAANAMARLRYDDLALLEQLEQRSLVLMGVPAEALPSASASSARHAAVAVGRQQQQEQQQQTRWAVKPDRRRRDRASPAAASASAGAASSSAAVSAAVEASPAGAAAEAAAPSAAGPMTASELLALVSAFGSLGYRPSQTWLLSFTRCTAPHLTTYASTPESLPTLLSSLGNTGHRPPPAWLQSACAAAAPHLPSYSSAQLKALAAGLCAMRHLPDDPWVAAYLGASAQLLPGYSAAELTVTISSLAGLGCRPGDEWMEGFYARATAVVGTTGGLTGPQAASILASLSKLNCRPTSDWLNTVLLGTRRSLSDASAQQLTELAASLARLRFRPPEPWLQQYFNASFQRLPFYTPAQACTAAQALARLGRRPTKLWMEEFGRLLGAKLPLMPGGQQCEAVAALVDLGYVPSPAWLAGYEQHSNAQLASCTSDQLCLVLPALAKVNFRPQVSWLYSFIMSAYSQLDAFSSPQLALVFECLPALTPHGSWLDEIIQICAAEAAMRGAGPVGSSSAAAVSAAEVAEPDAVVVPVPGIAAAAAAVAAAPAAAPAQPASAGLFATGPLYGDGPGASAAAGNGVASTAAVGPIDANVPINGAAAPTDAALSAAATLPDTSTIQISSELVPSSDAVTLSAPYRFSASAGYGSAASEAAANAAAAATVLDLTDMQLAAVPIATPAAAAAAAAAGGRAAEGMNGRREGRGGRSDSGWGGPGGGGGAGGGSVGRGDLSSPSADLLRRPARVPRPAVAVLASSIEAADAVPTSSASASAPNPAPSVTPSSVTAGSAISSPYAPASPTASWSDADLLLPGSPDQQPQLLAQPRPPAQLLQRPRLRSAFSALPASSPAMEPIVAAGSSAAAASSARLDAVAAATAARVAAAAASRRPLTSAAPLGLQAMDATAAAAMAAGSAVAAALAAGAADDEVRVQYLDSASLNRLIGPPDGPPGPDFSGCQKRAMEAAARKARRGTNNLFSLGRVWTNELGGSDAGPDIVGGTGGGMPSPVPSPVG

>RAT2 (EDP02536.1)

MQSPALAPSLPEPLPSAAALPDEPPLRSELIDLTRAVQRCRTWQQLRRLYWRVRTRQGPMNMVGFFTRLAALVGPGLPQLLGEEGEEEAIMVTAEGPGQELRVPGQEAEDGAAAGREAGAGKAQRSRSRGPEGSPRGQQGPPVPSGPLQSHAERAALRQLVVRMMYDACSFTREHAAADLLDVMRRNPLPPWNMPAFAAYTQQQVAALQAAHQRGGAGLGTRAGAGVGVGAGGGAAAGRQVSFLEWLREHPEMWEHPYEAILDEHVHHWRASPGAGAGTTGADGADSAYGSGRSRSSSGRSGAGGTRSRQAPVCYGPHELSALVWAAARLRLPGAVAAEHLTWLLDDLLSASFGVMPDFDGRQLSQLAYSLSQLGPLAVPPPPWRRAFLAASRRRLFQMDAQSLANLSHALEPLRLSPDPPWLRDLLAATGAAMSITGPGQGLRGQGQGHGGQGQEQGQQRWGPAQRPGTLRPAPPELTQLAWALFSLRRRCATRAATAAISDRGRSRPGSLEAVLRSWRKHYQCFHHQQRKHGVAAGDGGGVPQAMAAAAPATATTTTLGECPCCP

>ASA2 (EDP00850.1)

MRSAAVRVLGAQWAGVGAQEAGSRAARAFATATFVPGVSGDASGVVSAVDALMSHDSAATGKDVADAAVALAYLGTRGNRRVWGKVLEKAASTPLDGPSLANLSWALSAANVDHTRTLAELAGPLAASLKSLSPAQVSFAVEAVGKSGAADVELFAAVTELAAARTADFKAADLARLLWGFGAAGVQDGKLVKAASAGLVAKAAELGGREAAQALWGLAALRRVPDAALAGALTKALKAGVEAPADAAAAAWALATLAVKADAGTVKALADKAKAGVADLSAAQAVQGGWGLAMLGDKDGAAALLGAAAAAVQKDPTSLSPSALALLHAGAVVSGAGLPNPVSDFAAKGFGLAVEHGRHSRSSAAAAFHAELAEAVAYASGARHRPDVASKVASFVSSGPDGSTLDVVVPADANTKLAVLGVEAEALASSGAVLGGSLAAARVREAQGFKVAVVPQTEFPTGAPLKQRAAAVLGAIKKAVPGLSAMADKLSREL

>RAA8 (Cre10.g440000)

MLRVAGAGKFPGDANAEAATAAAARGFGGSRPGRGVRGSSGTGSRQGDGFGSVRGGRSGGRSWGGRSGSSGGRGPGSRGPPPQVGTRVPPASPDDVAAWVGTADGGDSGGGIMGRITAATLWQQLTAILESEAGAVAAAAAAAAATATATATAAAFPAAAATAAVGSMAASSTSTSIPPPGGDRAEEAAMPGEAGGGASTRTTPPPPPPSLGEPELLLLLWRAVCGCLQTFTDGSLLLLPPPPLPVGSQSGTGAALFPHMPALPGSTATTHAATPSPFGATMGGGGAIDDLFSFSASGVDLTNGLGTGAARAPLAAAGAAVTTVPMWTVAPLAGSGVARQRCLRALAAALARLAPRLSPAGLAAGVQLAARLGQVAAGGNTGGGGGGGAGGYVQEGHRQAAGAAAWELAAPLLQATAATAAAATAAAAVTAGAGDGGPALVAVAPETATALVDVLVALPLLQALEPDHGPGSHQQAGLQAQQQRRRPPQQQHDGQGDRPSEACPLSRRVLETALGTLCVRAGAVAPSTRELAVCLAAAARCGLAPPPAWLALLQCEVRSQLLLLTPAQGAVLLAAVQGLERHAGGSGGRGAAGMPAPLVAPAWADMVWREVRGRAGAAPAAGGGGGPSCGLGLGFGFSGGGVGGGVFGPQALVDPDLQELGWFADAGLLLMPPPPLPPSPSDTPQELGGQGGAGAGDGHGGREDPVGWLVQHLAVRTARQWLLPTEAAAPQAAGPGPQELVAVLAALARVAPFAGAAATAGKDAGGGATATDSAGAGADGGMVADAAATQLGGGAGWRQELLAAAVAAVLQCLGGTPALAAPAPAAQPATAELASRAGCSMEWLQLAQCTEALAALAQAAAEDEAQGNRAAAAVPTGPATAATAATAASAGSATAAAVGSAKLPDLDALVWRLQQALLEAAAAAGSRAAVATMAAAVGPGELVWLLRAEAALLRMQELWPRQAHHLPPARGLARRLLAALIAPLLRQAPLAQAAHNPASHAAAATAAAAMPPAQLLQLLAAAQHPWTAGMFDGELLAAGSSSNSSSSSSSSGRSTSRSGEAAAAGRLTVPAQAASARGVVVTALLDSLPLTTASAGARGDAAAAAAAAAALDLAVALLPAAVHALRTPADAVAALRAAADRQQRHQRQQQALQQQGQRTGRLRQADALAAAALPAALSRLLALAIPPPPHQEPGFMDVGAAAPAPAALVAPEVWCEALAALLDGVRALDPANAKAASSTSSPAAAVGAPAGPRRAATSSAAAAGYFAWPPQAFSPPPPPPPLVQLRRLWGALDRCWPLMAPGQLVRVLGLLADLHAPLSAMSSRGAAAAGGEPTAADLQALQRAVASRMADMTPTELAEAVTATARLPAAAPPCDEWLAALTTVLAAPPPPPAPLAGGLQGLRAAEALALVLACLDMKAAAPVTARDAVMVPVLPLLEQLGLALVADHGPPGPVRDAAKALRAAPDEAAAATAVAAAVAGAAAAPATPAAPSPPQPTRPLPPPPSCGELCRLLPRLAALATHPATAQAMGSAAAPMLSYLAAVGFAGAGPADLAACVSGLGRLLVLPGGHWVAALCEATRPLLLSRGTSLAELAALSHAMATALLLPPPQWQRDLEAATAQRMARAAAEAAQLAAAGVAEAPGSGRGGSRSAGGGGGGGGGLRSAADLKALDQLLTAQALWWRLSSGSSTSSSSSSSSSSSSSSSSSSSSSSSSSRKDSGAEAAAVTELRPSPGWLDALVECAAALMAALGPAASPQSALAGLPYTLHVLGHDPGPDFISALLAACSRYLPLLPLARDPGLAHEHTAPGAPTPTLSPTPNANPGGGGGGGTQQQQAQEAAGPHVVVQVPASAPAFTGPQLSRLALGLAGLGYQPEDAWVRCLQMESALRLSGCDPHELADLLEGLLLLAEGCEAGGGSSSSSASGSSSTSGSSGEAQWVDSRLVPAFLGCWWAAAEIAMHNDTAVAAAALERRARRVDLRLATAMRAELRARLSDRSGAGAAWGYWSEQLPGLDPDRLFEMSDQDIEAELAALQFPVFDAGMALAAAQEEQQADQQEQEEQEQEQGRTGRWRRPQPDGGSGGGGGPIKDGNKVSFDVGMPVLQRYTLWAILRGGGSSSTAAGEASEPAGRIPGPLPEGAAAWVGSLAVRSVALLSGPRAAPAADGRLTAAQLQQLAAAAAAPGSAVVVAPPAFWLESMLACQAAAMMLGAYGGAGSLSLAEYESAVGHDADGAEAGEPWRRQLLQRRAAAAQRAALLQAAAAQRAALLQAAAAARAWALPPVPEWGEAFLRASRLAFLSPPPPPLPQPSRQSSPPAAGVGPGASAADAADTAAAVTGPGGGDGSGGDGGSGGSASCLSELLQLLLAAVALDMQPGEGWLRAAEATSVDLMRGGGGSGVGPEEAVALVLALQRLGHTPSRLWAEAVLERMAQGLPTPTQLSEPGAPGAYATAAPSVTGNPAPMLGAAAAAAEAAGAAALLPPASVAGVVVALAAMRVRPSDGWVEAALAALHERHGDLPAPLRARLLAALPQLDVTPSAVWLGPAVEAFWEGVPLLLEPLRQRLRQLSRGGTETGAAAGAAAGAAGASSDTQPGLEAAGEGAGAPGDAAAAALVAPLVSGLTAVSELGVPVKQQQLTALLGRLSDLMAPPPTAAAAAAAAAAAAARSPGAATTSSAAATTTAAAATLAAAEVGRVLVCLAAMRRATPSRANQRVLDSLFAAAAERLPLSGPLTAELLDAWDKLVPP

**Figure S2**. OPR motif sequences used for the creation of Figures 4B and 4C.

**Name Start P-value Site**

CCS2 769 8.70E-018 RLSAFPPQELTTLLHALATLGHRPSGDWMAAAAGAVAA

TDA1 665 5.04E-017 KMYAASPLQLAAYVWALARLGYHPGDLWLYSLTQQLSR

RAA1 1452 9.30E-017 CLSQFDTEGLCRLLWALAAMDYVPERLWLRAVAGQLQA

TBC2 491 5.55E-016 RLTSFSPLQLAKAVQGLAALRYRPSPEWVEAYCSVLRP

CCS2 979 9.92E-016 GGSSCKAQDVCNALWAVAQLGLRPPRAWVLAVAAGALS

TDA1 977 6.76E-015 LLPGFNAKGLATVLQSMASLGIKPQRRWLLRALEALAD

TAA1 1002 8.43E-015 KLAAAPPQSLANLCWGLGKLGVKPPRAFVTAMAVASLG

TDA1 705 8.43E-015 RLAAASAGQLCEVLWALHRLGFSPDPDWRAQAAEAVAF

MBI1 856 1.17E-014 RLREFSDLELSNLVWALAVLKCQDRQLLVRAARVLVGR

MCG1 916 1.31E-014 ALPGFNAVDAAHSLWALAALGERPPPDWLQRLLVGVRG

MBI1 387 2.01E-014 RLTSALPQELSNLALGLAKLGYREVPLWAAIIAAGKAR

CCS2 1166 3.83E-014 ARGELGEQHCANVLWALAVLRYRPPPDVLRALGARAAQ

MBI1 460 5.26E-014 QLGAFTPSGLSNLLWACATAQCHVEELFDGAAAALLRV

RAP 369 8.88E-014 KVGEFNSQNVANIAGAFASMRHSAPELFAELSKRASTI

MCG1 992 2.03E-013 GLPRMEGQDFAMLLSGLVRLGGRPTPEWSARLMAALAP

TAB1 629 2.24E-013 RLPFYTPAQACTAAQALARLGRRPTKLWMEEFGRLLGA

TAB1 591 2.49E-013 SLSDASAQQLTELAASLARLRFRPPEPWLQQYFNASFQ

CCS2 1567 2.75E-013 SLPLLSPHEAATLASALVALRYAPPPLWLDRVEQLLLR

TBC2 415 4.12E-013 KLYECRPQDLANTLASVALLGLPPDADLRTSFYAAVRQ

MBI1 1227 5.03E-013 RLGRFCPWDASVVAWAYAKLDHPHRDLFESLQHHALRY

TDA1 1176 5.03E-013 LLDAMSGPQLARLVSALARVNFYPGREFLAAHSRATAR

RAA1 1414 8.27E-013 GGGGVGPRQLWGLAWSFARLGYAPSQEWMLALLSRAEA

CCS2 809 8.27E-013 AGGSMSPRQLSTTMWALAVLRQRPSRALMAAWALASLQ

RAA1 1490 9.13E-013 RARDFTPDQVVTLHCALARLGYAPRPEVCVALHAAAAR

MCG1 717 1.01E-012 VLPQCTPQGLANALSALAAAGHVPDAGWLERFYIESAA

TAB1 512 1.49E-012 LLPGYSAAELTVTISSLAGLGCRPGDEWMEGFYARATA

TAB1 705 1.64E-012 QLASCTSDQLCLVLPALAKVNFRPQVSWLYSFIMSAYS

TDA1 901 1.64E-012 KLASMSATHLTQALVSISQLRFVPPQSWLMAFLCASRA

RAP 406 1.99E-012 IINTFKGQEIAQLLWSFASLYEPADPLLESLDSAFKSS

CCS2 1529 2.66E-012 ARSSFPPRSLPVLLWSLSRLGCRPPEECMRRLLVHSVE

MCG1 1030 3.54E-^012^ RLRRLRLRSLSQCVWALYRLRVQPPPEVLTQLAAELRR

MCG1 954 3.54E-012 ALVAAPPSELAVLLWSLSELRFRPTWSWIADCVEASAP

RAA1 1763 3.54E-012 QLGQAATADLSTTIAALADLQYRPSDQWMALFTAEARR

TBC2 732 4.28E-012 LKNRCTDLHLANLAGSLAAWGVRPDGRWAARLMWRSQV

TBC2 453 4.28E-012 QQRRFGPRELATTLWAYGAMGTYVQEDAVQLVLELSRA

RAT2 395 5.18E-012 RLFQMDAQSLANLSHALEPLRLSPDPPWLRDLLAATGA

TBC2 812 1.09E-011 SQPAFEPHHYGTLMASLHALGIQPPQEWLTRMLLSTYR

RAA8 1851 1.44E-011 SAPAFTGPQLSRLALGLAGLGYQPEDAWVRCLQMESAL

TBC2 653 1.90E-011 QLDKLDAQQLAGVLNSLAAQQYRPQPQMQEVVLAATQA

RAP 330 2.49E-011 CLPECSAQGISNISWALSKIGGELLYLTEMDRVAEVAT

MBI1 818 3.57E-011 QVDRLDPQGLSTLLWAFARAGKHYHGPLTTTICRVAAP

TBC2 850 4.67E-011 CWDRFSVTHWSSLLPALVLLKARPPREWLRRFEATSAA

TAB1 667 6.65E-011 KLPLMPGGQQCEAVAALVDLGYVPSPAWLAGYEQHSNA

MBI1 498 6.65E-011 PPAQLNSQDVANTAWAFAKVQHPHPGLMRHLGGLVLRA

TBC2 772 7.27E-011 NEDRMSPRALVALLQAMVSLGLSPNPVWTQLCLQAAVR

CCS2 1126 7.93E-011 GAQRWRAGDVAGMMWALAKLRVRPPPGQMLRLCRAAAG

TAB1 553 8.66E-011 TTGGLTGPQAASILASLSKLNCRPTSDWLNTVLLGTRR

TAB1 396 1.03E-010 AAGPMTASELLALVSAFGSLGYRPSQTWLLSFTRCTAP

MBI1 1190 1.46E-010 LMHLLTPHEVVQVLWSVAYHSHSCPELLDAAAPAIASR

MCG1 506 1.59E-010 HLHRFNAQDLSLVALALAALRGRDGGVSSGSGSGEGST

RAP 473 1.59E-010 PALSFNRDQLGNIAWSYAVLGQVERPFFANIWNTLTTL

ASA2 165 1.73E-010 RTADFKAADLARLLWGFGAAGVQDGKLVKAASAGLVAK

TAB1 474 1.89E-010 HLPSYSSAQLKALAAGLCAMRHLPDDPWVAAYLGASAQ

MBI1 299 4.39E-010 QQQYPSARQFANVVWALGSMRIRSPRQRPDTPPASSPS

MBI1 424 6.66E-010 RLPAFKPQELHNLAWAVAAASQDRSMISAAVQAALPQL

MCG1 468 1.00E-009 RAAAFRPKDISLSLWALSKLAARPPAGGLQLLLAAAEP

TAB1 436 1.09E-009 TTYASTPESLPTLLSSLGNTGHRPPPAWLQSACAAAAP

MBI1 1264 1.09E-009 YGSRYKEPCLLRLVWACAQLQLHVREPLLAQLHALRTG

TAA1 1711 1.09E-009 ASEPWAPVDVFKALWACAKMNRHPGPQILAAAERSWVL

TBC2 1038 1.09E-009 LRVRYAPSELVLTAGWLGSLGLRPPPEWLQACAEVAAR

RAA8 2394 2.07E-009 GGSGVGPEEAVALVLALQRLGHTPSRLWAEAVLERMAQ

RAA1 1162 2.24E-009 HVSAASGSDVAQISYGMGALQITCPQLYGAVLHVSASQ

RAA1 1686 2.84E-009 VAATFDATCVSQSLWALAELRETPGLPHSGAAAAAAAA

ASA2 202 3.07E-009 KAAELGGREAAQALWGLAALRRVPDAALAGALTKALKA

TAA1 1040 3.07E-009 QLPHFTPQELATTAFVLATWGGRLGAAASGLVRHVVAT

MBI1 697 3.89E-009 RAASMSDQELANALWAAGELRGAGHYVPPAAVAPLFAA

TDA1 863 4.20E-009 HLRGLRRVHLLRLLLALAESGHRPREELMARVLGCLQP

TBC2 615 4.91E-009 AAGGFSGGELQQLLEGLTRLALQPPLEWMQAFVAALQP

TBC2 529 4.91E-009 ALRRMSSRELCAVLLALASLQVGLDGGTRAALLVHTFS

MCG1 790 5.30E-009 SSRWCNADDLAHMAAAAAELRLAPPRWWAARLYGAMER

TBC2 693 5.30E-009 KQLLADTTCSAALLTALRRLNIEPPPGWVGALLEESRS

MBI1 658 6.19E-009 RRRDLTPQGASNALWAAGRLQPCPVPPDALADALRAAS

TDA1 939 6.19E-009 QLRHYTPAHVTATYQVFAGWQLRPPRSYLEALHTYMDG

RAA8 2475 7.21E-009 AAALLPPASVAGVVVALAAMRVRPSDGWVEAALAALHE

MBI1 1153 1.05E-008 PTAQGHAKSMAKLLWGFAKCNLYNQALYRLLVQELRPL

MBI1 571 1.05E-008 GLRGVATQELVNVLWAFASMPPQGLGLPDAAAGGVPAQ

RAT2 355 1.42E-008 VMPDFDGRQLSQLAYSLSQLGPLAVPPPPWRRAFLAAS

TAB1 272 1.42E-008 SCGVMDGRGVATAANAMARLRYDDLALLEQLEQRSLVL

MBI1 162 1.53E-008 HKSQFNSIHTAAAIVKLAKLTAGEPEQPHHQQQHQRHQ

CCS2 847 2.06E-008 TMAHASAYDVSQSLWAVAKLHRDAVMDAAAAAEAGGGG

TDA1 627 2.06E-008 DLFLATPAQLCRLASSLPLLRLAPSSRWLDHFAAVSRS

TBC2 568 2.77E-008 PLPGMAPGEVALSLWALGRLSAVDMDLPALIDLDMSGR

TAA1 782 3.20E-008 LGLSMSPAEAVGVLRACAALKWAPSVLFSELLLPLLRQ

MCG1 832 3.97E-008 GGGGCSARQLSQMLHGAAQLHRSYGQLPLAPVASNTKS

CCS2 725 4.27E-008 AEPRLGPRQLATCLGALARLRARGWHVASERRLLHLSV

RAA8 522 4.93E-008 GAVAPSTRELAVCLAAAARCGLAPPPAWLALLQCEVRS

RAP 162 5.68E-008 KKKKKKKSKKVIVKKDKVKSKSIPEDDFDTEDEDLDFE

TBC2 346 6.55E-008 GLDSLSGPQLAAVFTGLAVLRRPRQQQQQQQQAGAAGA

TAA1 925 7.54E-008 EGALLGAEDMTQLLWVCVALRYRGGAVLRPLLQLLLLV

RAA8 1544 8.09E-008 GFAGAGPADLAACVSGLGRLLVLPGGHWVAALCEATRP

RAA8 1348 9.30E-008 RMADMTPTELAEAVTATARLPAAAPPCDEWLAALTTVL

TAA1 1243 9.97E-008 CQAILGPQALAGVLSAMSDLGHYPAGWAARGLLRCVRL

CCS2 1244 1.07E-007 DVAGAGPQLVSTALWCCLRLGLPPGRGLLLPLLRAAAA

ASA2 128 1.15E-007 SLKSLSPAQVSFAVEAVGKSGAADVELFAAVTELAAAR

ASA2 55 1.23E-007 HDSAATGKDVADAAVALAYLGTRGNRRVWGKVLEKAAS

TAA1 1281 1.41E-007 GLVEADATEAAAIVAALAAWGERLEEGEAAAAAAAAAA

TAB1 743 1.51E-007 QLDAFSSPQLALVFECLPALTPHGSWLDEIIQICAAEA

TAA1 1486 2.27E-007 PAGRRRVPALTTLLWAMASLRQDVPELLDDLQAALMGL

RAA1 1632 2.27E-007 AAAAVDGSDLSMALWALALLRRQQQQQQQQPAADVLQL

RAT2 469 4.12E-007 GTLRPAPPELTQLAWALFSLRRRCATRAATAAISDRGR

RAA8 1889 6.09E-007 RLSGCDPHELADLLEGLLLLAEGCEAGGGSSSSSASGS

RAA1 1817 6.92E-007 TAATATNEDHGLIAYGLAVLGWPLSEAWVQELAAGGYR

RAP 560 8.38E-007 KTKRFNQKITSSFQKEVGRLLISTGLDWAKEHDVDGYT

MBI1 777 8.93E-007 GLGSLGPQELCVLAAAVAEAVHVAAYCNPILLNGLANA

RAP 281 1.22E-006 SPSPLSPLNIATALHRIAKNMEKVSMMRTRRLAFARQR

RAA8 2351 1.39E-006 GGSASCLSELLQLLLAAVALDMQPGEGWLRAAEATSVD

TAA1 1445 1.39E-006 AQPSIEPEVALSLLRSLARLRWHSDPLEGALVRAAAAL

RAA8 1584 2.89E-006 LSRGTSLAELAALSHAMATALLLPPPQWQRDLEAATAQ

TAA1 2011 2.89E-006 SCYEWDRISHQDVWTKMVYLQAKIDRRTGTGLAASSVS

RAA8 2513 3.89E-006 RHGDLPAPLRARLLAALPQLDVTPSAVWLGPAVEAFWE

RAA8 1295 4.37E-006 CWPLMAPGQLVRVLGLLADLHAPLSAMSSRGAAAAGGE

ASA2 238 1.37E-005 KAGVEAPADAAAAAWALATLAVKADAGTVKALADKAKA

RAP 201 2.24E-005 GFVEDKMGDLRKRVSSLAGGMFEEKKEKMKEQLAQRLS

RAT2 165 8.99E-005 MYDACSFTREHAAADLLDVMRRNPLPPWNMPAFAAYTQ

RAP 25 1.85E-004 HNHRNLHISLSSSSFASGILPLSNKKYRFVGPLAQRSS

RAP 240 2.23E-004 FSGPSDRMKEINLNKAIIEAQTAEEVLEVTAETIMAVA

RAP 107 2.33E-004 DPLDIQPPKKRKKQKNSKALEDTEGMDWCVRARKIALK
